# Fine Tuning the Hydrophobicity of a New Three-Dimensional Cu^2+^ MOF through Single Crystal Coordinating Ligand Exchange Transformations

**DOI:** 10.1021/acs.inorgchem.3c04060

**Published:** 2024-02-09

**Authors:** Nikos Panagiotou, Dimitrios A. Evangelou, Manolis J. Manos, John C. Plakatouras, Anastasios J. Tasiopoulos

**Affiliations:** † Department of Chemistry, University of Cyprus, 1678 Nicosia, Cyprus; ‡ Department of Chemistry, University of Ioannina, 45110 Ioannina, Greece

## Abstract

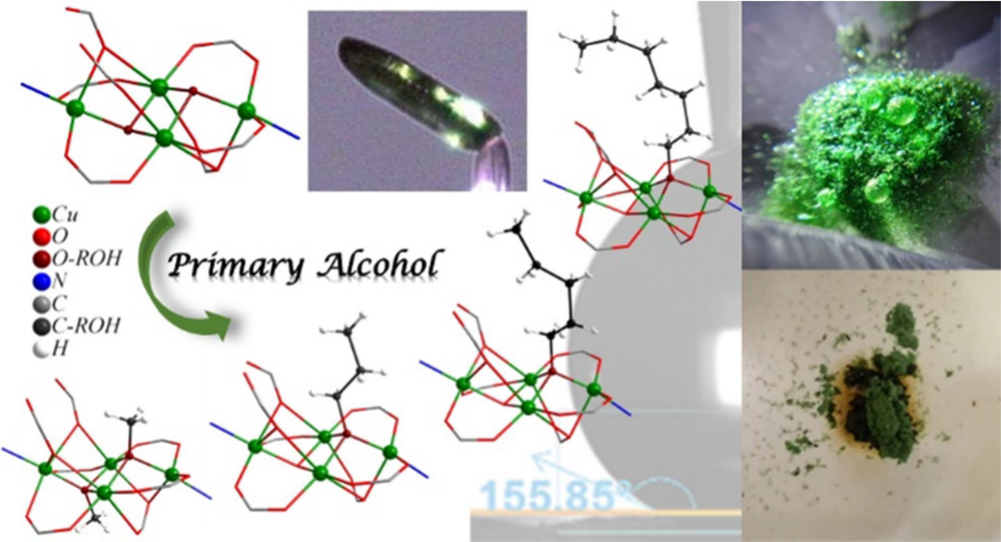

The synthesis, characterization, and single–crystal–to–single–crystal (SCSC) exchange reactions of a new 3D Cu^2+^ MOF based on 5-aminoisophthalic acid (H_2_AIP), [Cu_6_(μ_3_-ΟΗ)_3_(ΑΙΡ)_4_(HΑΙΡ)]_*n*_·6*n*DMF·*n*H_2_O - **UCY-16**·6*n*DMF·*n*H_2_O, are reported. It exhibits a 3D structure based on two [Cu_4_(μ_3_–OH)_2_]^6+^ butterfly–like secondary building units, differing in their peripheral ligation, bridged through HAIP^–^/AIP^2–^ ligands. This compound displays the capability to exchange the coordinating ligand(s) and/or guest solvent molecules through SCSC reactions. Interestingly, heterogeneous reactions of single crystals of **UCY-16**·6*n*DMF·*n*H_2_O with primary alcohols resulted not only in the removal of the lattice DMF molecules but also in an unprecedented structural alteration that involved the complete or partial replacement of the monoatomic bridging μ_3_–OH^–^ anion(s) of the [Cu_4_(μ_3_–OH)_2_]^6+^ butterfly structural core by various alkoxy groups. Similar crystal-to-crystal exchange reactions of **UCY-16**·6*n*DMF·*n*H_2_O with long-chain aliphatic alcohols (C_*x*_H_2*x*+1_OH, *x* = 8–10, 12, 14, and 16) led to analogues containing fatty alcohols. Notably, the exchanged products with the bulkier alcohols **UCY-16/***n*-C_*x*_H_2*x*+1_OH·S′ (*x* = 6–10, 12, 14, and 16) do not mix with H_2_O being quite stable in this solvent, in contrast to the pristine MOF, and exhibit a hydrophobic/superhydrophobic surface as confirmed from the investigation of their water contact angles and capability to remove hydrophobic pollutants from aqueous media.

## Abstract 2

A 3D Cu^2+^ MOF is reported with a capability to exchange through single-crystal-to-single-crystal (SCSC) transformation reactions, with various primary alcohols (C_*x*_H_2*x*+1_OH, *x* = 1−7), the bridging μ_3_−OH^−^ anion(s) of its secondary building units, by μ_3_-OR^−^ groups. These SCSC exchange reactions as well as crystal-to-crystal ones with long-chain aliphatic alcohols (C_*x*_H_2*x*+1_OH, *x* = 8−10, 12, 14, and 16) led to analogues displaying hydrophobic/superhydrophobic surfaces, high stability in water, and affinity for hydrophobic pollutants.

## Introduction

During the past couple of decades, materials based on metal organic frameworks (MOFs) are continuously being considered for several applications.^1^ Gas storage/separation,^2−5^ catalysis,^6,7^ sensing,^8,9^ removal of pollutants from the environment,^10−12^ and water harvesting^13−15^ are among the most prominent applications of MOFs in areas of global interest.

The exploding progress in the synthesis of functional MOFs allows the design of materials exhibiting the desired characteristics for targeted application. For example, by utilizing the molecular building block (MBB) approach, MOFs with varying network topologies and structural characteristics can be readily designed and synthesized^16^ giving rise to materials with a plethora of different metal ions, organic ligands, secondary building units (SBUs), functional groups, network topologies, and so forth. Among the various building blocks appearing in MOF chemistry, the Cu^2+^ paddle - wheel secondary building unit (SBU) consisting of two Cu^2+^ ions adopting a square pyramidal coordination geometry is one of the most common ones.^17^ In fact, some of the most well–known MOFs including HKUST-1 ([Cu_3_(btc)_2_(H_2_O)_3_]; btc^3–^: 1,3,5-benzenetricarboxylate) are based on this dinuclear SBU.^1,18^ For this reason, several groups^19−23^ including ours^24,25^ have been involved in the synthesis of Cu^2+^ paddle wheel-based MOFs exhibiting interesting structural characteristics and promising gas sorption properties. However, it is well–known that Cu^2+^ MOFs including HKUST-1^18,26−28^ as well as other MOFs suffer from limited hydrolytic stability. One method to enhance the hydrolytic stability of MOFs is to increase the hydrophobicity of their pore surface and/or external crystal surface.^29−33^ Such hydrophobic MOFs have attracted significant interest not only because of their stability in water but also due to their diverse potential applications including humid CO_2_ capture,^34−36^ alcohol/water, organic molecules/water and oil/water separations,^37,38^ removal of pollutants from air or water,^39,40^ substrate-selective catalysis,^41^ and anticorrosion/self-cleaning coatings.^42,43^ As a result of this interest, several synthetic methods have been reported that lead to MOFs with enhanced hydrophobicity. These synthetic methods include the de novo synthesis of MOFs using ligands containing hydrophobic functional groups^29,30,32,34,35,39−42,44,45^ and also postsynthesis modifications (PSM)^29,31,33,46−50^ of well–known MOFs. Some examples of the latter include external coating with octadecylamine on MIL-101(Cr), UiO-66, HKUST-1, and ZIF-67 MOFs^31^ and functionalization with aliphatic alkane groups in IRMOF-3 and MIL-53(Al)–NH_2_ through amide bond formation upon reaction of the −NH_2_ group of the 2-amino-1,4-benzenedicarboxylate ligand with various alkyl anhydrides.^33^ Moreover, the fabrication of MOF-based composites is another PSM strategy to encapsulate and immobilize MOF particles in a hydrophobic matrix.^51,52^

Single–crystal–to–single–crystal (SCSC) transformations are a subcategory of the PSM method that has attracted a significant interest because it allows the acquisition of direct structural information for the achieved modifications through single crystal X-ray crystallography. Such transformations can take place from the heterogeneous reactions of single crystals of a MOF with chemical species being in the liquid or gas phase or from their exposure to an external stimuli and can lead to the targeted modification of their structure^53−61^ and the fine-tuning of their properties.^55,56,59^ Several SCSC transformations have been reported in the literature which can be summarized in the following three groups: (a) insertion/removal of guest molecules,^57,58,60,62−66^ (b) modifications of the parent structure including the coordination mode of organic ligands, or metal ion exchange (transmetalation)/insertion,^59,60,67−69^ and (c) changes in the coordination environment of the metal ions.^53−56,67,68,70,71^ Interestingly, such SCSC transformations have allowed not only the improvement of the magnetic,^60,61^ photoluminescence,^55^ sorption,^62,63,69,72,73^ and catalytic^73,74^ properties of MOFs but also the elucidation of the exact mechanisms of these processes. Although significant progress has been realized in the investigation of a series of SCSC transformations of various types, such exchange reactions leading to the increase of the hydrophobicity of MOFs are very uncommon.

We herein report a new 3D Cu^2+^ MOF based on 5-aminoisophthalic acid (H_2_AIP) and tetranuclear, butterfly-like SBUs with the molecular formula [Cu_6_(μ_3_-ΟΗ)_3_(ΑΙΡ)_4_(ΗΑΙΡ)]_*n*_·6*n*DMF·*n*H_2_O - **UCY-16**·6*n*DMF·*n*H_2_O. This compound exhibited exceptional SCSC transformation properties that involved either the exchange of only guest solvent molecules or both guest solvents and coordinating ligand(s). The latter took place from heterogeneous reactions of single crystals of **UCY-16**·6*n*DMF·*n*H_2_O with normal and primary alcohols which led to the complete/partial replacement of the guest solvent molecules and the monoatomic μ_3_-ΟΗ^–^ anion(s) of the [Cu_4_(μ_3_–OH)_2_]^6+^ butterfly–like core by the corresponding alcohol/alkoxy groups leading to **UCY-16**/*n*-C_*x*_H_2*x*+1_OH·S′ (*x* = 1, 2, 3, 4, 5, 6, and 7; S′ = lattice solvents). Interestingly, such SCSC transformations involving a triply bridging anion of the structural core of the SBU of a MOF have not been observed previously. Crystal-to-crystal exchange reactions involving long-chain aliphatic alcohols (C_*x*_H_2*x*+1_OH, *x* = 8–10, 12, 14, and 16) were also investigated resulting in analogues containing fatty alcohols. The synthesized exchanged analogues display hydrophobic/superhydrophobic surfaces as indicated by the determination of their water contact angles and high stability in water, in contrast to the pristine MOF which is unstable in water. Overall, the reported SCSC transformations provide an alternative, unprecedented strategy for fine-tuning the hydrophobicity of MOFs.

## Experimental Section

### Materials

Reagent grade chemicals were obtained from commercial sources (Aldrich, Merck, Alfa Aesar, TCI, etc.) and used without further purification. All synthetic procedures were carried out in air.

Synthesis

#### Synthesis of [Cu_6_(μ_3_-ΟΗ)_3_(ΑΙΡ)_4_(ΗΑΙΡ)]_*n*_·6*n*DMF·*n*H_2_O - **UCY-16**·6*n*DMF·*n*H_2_O

Solid Cu(NO_3_)_2_·2.5H_2_O (0.08g, 0.34 mmol) was added in one portion to a clear solution of H_2_AIP (0.1 g, 0.55 mmol) in DMF/H_2_O (8 mL/2 mL) in a 20 mL glass vial, and the reaction mixture was sonicated until complete dissolution of the reactants. The vial was sealed, placed in an oven at 100 °C, and left undisturbed for ∼4 to 6 h. Then, it was cooled to room temperature, and X-ray quality green plates of **UCY-16**·6*n*DMF·*n*H_2_O were isolated by filtration, washed with DMF (3 × 5 mL), and dried in air. The reaction yield was ∼82% based on Cu(NO_3_)_2_·2.5H_2_O. Anal. Calcd **UCY-16**·6*n*DMF·*n*H_2_O: C_58_H_73_N_11_O_30_Cu_6_, C 39.02, H 4.12, N 8.63; Found: C 38.87, H 4.27, N 8.39.

#### Preparation of **UCY-16**/S (S = Benzene (Bz), Toluene (Tol), Chlorobenzene (PhCl))

In 5 mL of the corresponding solvent, in a 23 mL Teflon lined Parr acid digestion bomb, were added single crystals of the pristine **UCY-16**·6*n*DMF·*n*H_2_O (0.1 g, 0.075 mmol). The bomb was sealed, placed in an oven at 100 °C, left undisturbed for 3 days for Bz and Tol and 5 days for PhCl and then was removed from the oven and remained for ∼3 h at room temperature to cool down. The crystals of the corresponding modified product, **UCY-16**/S, were isolated by filtration and dried in air or placed in the corresponding pure solvent for further studies. Anal. Calcd: **UCY-16**/Bz: (**UCY-16**·3Bz·DMF·2H_2_O C_61_H_58_N_6_O_26_Cu_6_), Calc.: C 43.81, H 3.50, N 5.02; Found: C 43.52, H 3.63, N 5.19. **UCY-16**/Tol: (**UCY-16**·3Tol·DMF·2H_2_O C_64_H_64_N_6_O_26_Cu_6_), Calc.: C 44.94, H 3.54, N 4.91; Found: C 44.67, H 3.71, N 4.63. **UCY-16**/PhCl: (**UCY-16**·3PhCl·H_2_O C_58_H_46_N_5_O_24_Cl_3_Cu_6_), Calc.: C 41.35, H 2.75, N 4.16; Found: C 41.65, H 2.87, N 4.34.

#### Preparation of **UCY-16**/MeCN

Single crystals of the pristine **UCY-16**·6*n*DMF·*n*H_2_O (0.1 g, 0.075 mmol) were suspended in 5 mL of MeCN in a glass vial. The vial was left at room temperature for 7 days. The solvent was decanted and exchanged with fresh solvent (5 mL) every day. The crystals of the modified product, **UCY-16**/MeCN, were isolated by filtration, dried in air, or placed in MeCN for further studies. Anal. Calcd: **UCY-16/**MeCN: (**UCY-16**·6MeCN·2H_2_O: C_52_H_51_N_11_O_25_Cu_6_), Calc.: C 38.76, H 3.19, N 9.56; Found: C 38.84, H 3.36, N 9.73.

#### Preparation of (**UCY-16**/*n*-C_*x*_H_2*x*+1_OH)·S′ (*x* = 1, 2, 3, 4, 5, 6, 7; S′ = Lattice Solvents)

In 10 mL of the corresponding primary alcohol, to a 23 mL Teflon lined Parr acid digestion bomb, were added single crystals of the pristine **UCY-16**·6*n*DMF·*n*H_2_O (0.1 g, 0.075 mmol). The bomb was sealed and placed in an oven at 60 °C for 2, 3, and 5 days for CH_3_OH, C_2_H_5_OH, and *n*-C_3_H_7_OH, respectively, at 100 °C for 4 and 5 days for *n*-C_4_H_9_OH and *n*-C_5_H_11_OH, respectively, at 130 °C for 7 days for *n*-C_6_H_13_OH and at 150 °C for 7 days for *n*-C_7_H_15_OH and then was removed from the oven and kept for ∼3 h at room temperature to cool down. The crystals of the corresponding modified product, (**UCY-16**/*n*-C_*x*_H_2*x*+1_OH)·S′ (*x* = 1, 2, 3, 4, 5, 6, 7; S′ = lattice solvents), were isolated by filtration, washed with the corresponding primary alcohol, and dried in air or placed in the corresponding pure solvent for further studies. Anal. Calcd: **UCY-16**/CH_3_OH: ((**UCY-16**/CH_3_OH)·7CH_3_OH·3.5H_2_O, C_49.5_H_69_N_5_O_33.5_Cu_6_), Calc.: C 36.00, H 4.21, N 4.24; Found: C 36.13, H 4.04, N 4.33; **UCY-16**/C_2_H_5_OH: ((**UCY-16**/C_2_H_5_OH)·7C_2_H_5_OH·2H_2_O, C_56_H_79_N_5_O_32_Cu_6_), Calc.: C 39.21, H 4.64, N 4.08; Found: C 39.37, H 4.81, N 3.95; **UCY-16**/*n*-C_3_H_7_OH: ((**UCY-16**/*n*-C_3_H_7_OH)·5*n*-C_3_H_7_OH, C_58_H_75_N_5_O_28_Cu_6_), Calc: C 41.68, H 4.52, N 4.19; Found: C 41.83, H 4.89, N 4.36; **UCY-16**/*n*-C_4_H_9_OH: ((**UCY-16**/*n*-C_4_H_9_OH)·5*n*-C_4_H_9_OH·0.5DMF, C_63.5_H_86.5_N_5.5_O_28.5_Cu_6_), Calc.: C 43.23, H 4.94, N 4.37; Found: C 43.46, H 4.79, N 4.64; **UCY-16**/*n*-C_5_H_11_OH: ((**UCY-16**/*n*-C_5_H_11_OH)·5*n*-C_5_H_11_OH, C_70_H_99_N_5_O_28_Cu_6_), Calc: C 45.70, H 5.42, N 3.81; Found: C 45.47, H 5.72, N 4.05; **UCY-16**/*n*-C_6_H_13_OH: ((**UCY-16**/*n*-C_6_H_13_OH)·3*n*-C_6_H_13_OH, C_64_H_83_N_5_O_26_Cu_6_), Calc: C 44.70, H 4.86, N 4.07; Found: C 44.58, H 4.72, N 4.18; **UCY-16**/*n*-C_7_H_15_OH: ((**UCY-16**/*n*-C_7_H_15_OH)·3*n*-C_7_H_15_OH, C_68_H_91_N_5_O_26_Cu_6_), Calc: C 45.99, H 5.17, N 3.94; Found: C 46.17, H 5.06, N 3.89.

#### Preparation of **UCY-16**/*n*-C_*x*_H_2*x*+1_OH·S′ (*x* = 8–10, 12, 14, and 16; S′: Lattice Solvents)

In 15 mL of the corresponding liquid primary (*n* = 8–10) alcohol or 15g of the corresponding solid primary alcohol (*n* = 12, 14, and 16), in a 23 mL Teflon lined Parr acid digestion bomb, were added single crystals of the pristine **UCY-16**·6*n*DMF·*n*H_2_O (0.1 g, 0.075 mmol). The bomb was sealed and placed in an oven operating at 150 °C, left undisturbed for 10 days, and then was removed from the oven and left at room temperature for 4 h to cool down. The crystals of the corresponding modified product, **UCY-16**/*n*-C_*x*_H_2*x*+1_OH (*x* = 8–10, 12, 14 and 16), were isolated by filtration, washed with the corresponding liquid primary alcohol and hexane or only with hexane (in the case of solid primary alcohols), and dried in air. Anal. Calcd: **UCY-16**/*n*-C_8_H_17_OH: ((**UCY-16**/*n*-C_8_H_17_OH)·2*n*-C_8_H_17_OH, C_64_H_81_N_5_O_25_Cu_6_), Calc: C 45.17, H 4.80, N 4.12; Found: C 44.85, H 4.92, N 4.01; **UCY-16**/*n*-C_9_H_19_OH: ((**UCY-16**/*n*-C_9_H_19_OH)·2*n*-C_9_H_19_OH, C_67_H_87_N_5_O_25_Cu_6_), Calc: C 46.15, H 5.03, N 4.02; Found: C 46.32, H 5.26, N 3.97; **UCY-16**/*n*-C_10_H_21_OH: ((**UCY-16**/*n*-C_10_H_21_OH)·1.5*n*-C_10_H_21_OH·DMF, C_68_H_89_N_6_O_25.5_Cu_6_), Calc: C 45.89, H 5.04, N 4.72; Found: C 46.05, H 5.17, N 4.69; **UCY-16**/*n*-C_12_H_25_OH: ((**UCY-16**/*n*-C_12_H_25_OH)·1.5*n*-C_12_H_25_OH·DMF, C_73_H_99_N_6_O_25.5_Cu_6_), Calc: C 47.40, H 5.39, N 4.54; Found: C 47.57, H 5.63, N 4.49; **UCY-16**/*n*-C_14_H_29_OH: ((**UCY-16**/*n*-C_14_H_29_OH)·1*n*-C_14_H_29_OH·DMF, C_71_H_94_N_6_O_25_Cu_6_), Calc: C 47.04, H 5.23, N 4.64; Found: C 47.32, H 5.01, N 4.89; **UCY-16**/*n*-C_16_H_33_OH: ((**UCY-16**/*n*-C_16_H_33_OH)·1*n*-C_16_H_33_OH·DMF, C_75_H_102_N_6_O_25_Cu_6_), Calc: C 48.20, H 5.50, N 4.50; Found: C 48.36, H 5.78, N 4.29.

#### Preparation of MOF Films for Water Contact Angle Measurements

30 mg of **UCY-16**/*n*-C_*x*_H_2*x*+1_OH·S′ (*x* = 6, 8–10, 12, 14, and 16) was dispersed in 1.5 mL of CH_2_Cl_2_ in a glass vial. The mixture was subjected to ultrasonication for 10 min and kept under stirring. A small portion of the resulting suspension was spread on a microscope coverslip using a Pasteur pipet and dried in air. This step was repeated several times until the slip was covered with a dense film of the MOF.

#### Isolation of the Magnetic superhydrophobic Composite **UCY-16**/*n*-C_16_H_33_OH-Fe_3_O_4_

90 mg of **UCY-16**/*n*-C_16_H_33_OH and 30 mg of Fe_3_O_4_ were mixed in a 10 mL glass vial containing 4 mL of acetone. The mixture was stirred for 15 min, and the magnetic composite was isolated via centrifugation and dried in the air.

### Stability Tests

Stability in organic solvents was investigated as follows: in 10 mL of the corresponding organic solvent were suspended 50 mg of the MOF and the mixture was stirred for ∼72 h, while the solvent was replenished every 24 h. The powder was then isolated by filtration and analyzed by pXRD. Stability in aqueous solutions was investigated following the below procedure: 50 mg of each compound was added in 5 mL of water and the suspension was stirred for 24 h. The powder was then isolated by filtration and analyzed by pXRD.

### Physical Measurements

Elemental analyses (C, H, and N) were performed by the in-house facilities of the University of Cyprus, Chemistry Department. IR spectra were recorded on ATR in the 4000–700 cm^–1^ range using a Shimadzu Prestige −21 spectrometer. pXRD patterns were recorded on a Shimazdu 6000 Series X-ray diffractometer (Cu Kα radiation, λ = 1.5418 Å). Thermal stability studies were performed with a Shimadzu TGA 50 thermogravimetric analyzer. Water contact angles were determined from digital images obtained with the use of smartphone equipped with macro-lens, by using the drop shape analysis utility of the ImageJ software, particularly the low-bond axisymmetric drop shape analysis (LBADSA) method.^75,76^ The contact angles were further verified using an Attension Theta Flex (Biolin Scientific) contact angle meter.

### Single Crystal X-ray Crystallography

Single crystal X-ray diffraction data were collected on a Rigaku Supernova A diffractometer equipped with a CCD area detector utilizing Cu–Kα (λ = 1.5418 Å) radiation. A suitable crystal was mounted on a Hampton cryoloop with Paratone-N oil and transferred to a goniostat, where it was cooled for data collection. The structures were solved by direct methods using SHELXT and refined on F^2^ using full-matrix least-squares using SHELXL14.1.^77^ Software packages used are as follows: CrysAlis CCD for data collection, CrysAlis RED for cell refinement and data reduction,^78^ WINGX/Olex2 for geometric calculations,^79,80^ and DIAMOND for molecular graphics.^81^ The non-H atoms were treated anisotropically, whereas the aromatic hydrogen atoms were placed in calculated, ideal positions and refined as riding on their respective carbon atoms. Several restraints (DFIX, SIMU, RIGU, and DELU) were used to fix the thermal ellipsoids and geometry of the aliphatic alcohols and the corresponding bridging alkoxy groups. Electron density contributions from disordered guest molecules were handled using the SQUEEZE procedure from the PLATON software suit.^82^ Selected crystal data for the pristine MOF **UCY-16**·6*n*DMF·*n*H_2_O and the exchanged analogues are summarized in Table S1 in SI. CCDC 2301078–2301089 contain the supplementary crystallographic data for this paper.

## Results and Discussion

### Structure Description

Compound **UCY-16**·6*n*DMF·*n*H_2_O was synthesized under solvothermal conditions from the reaction of Cu(NO_3_)_2_·2.5H_2_O with H_2_AIP in DMF/H_2_O (8/2 mL) in a 1: ∼ 1.6 molar ratio at 100 °C. **UCY-16**·6*n*DMF·*n*H_2_O was obtained as light green bladed crystals in ∼82% yield based on Cu(NO_3_)_2_·2.5H_2_O. The isolation of compound **UCY-16**·6*n*DMF·*n*H_2_O highlights the ability of the widely used, in Cu^2+^ MOF chemistry,^83−85^ligand H_2_AIP to afford new compounds, probably due to its significant bridging capability (vide infra). Structure elucidation of compound **UCY-16**·6*n*DMF·*n*H_2_O revealed that it crystallizes in the orthorhombic *Pbca* space group.

**Figure 1:**
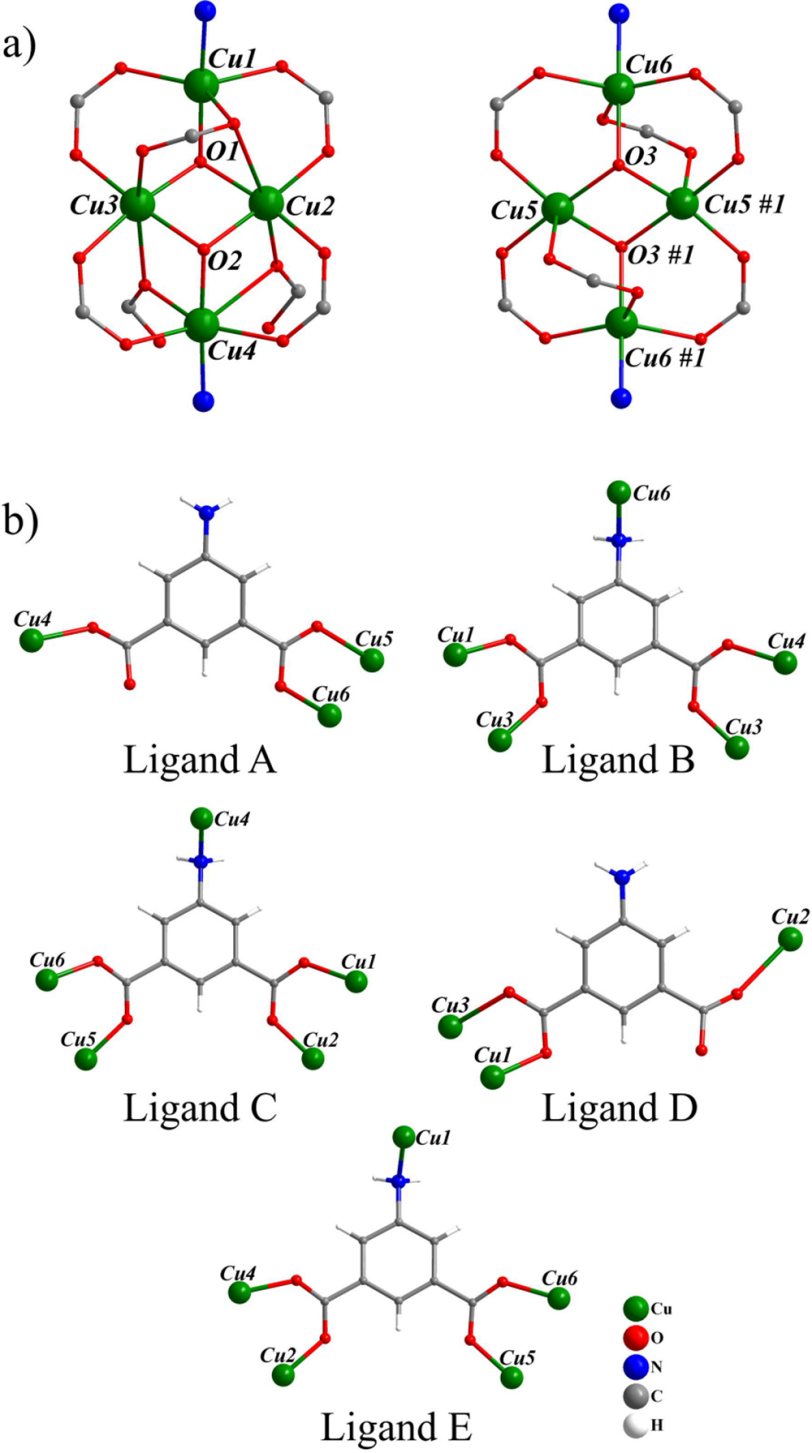
(a) [Cu_4_(μ_3_–OH)_2_(COO)_7/6_(NH_2_)_2_]^−1/0^ butterfly–like SBUs of **UCY-16**·6*n*DMF·*n*H_2_O. Symmetry operation to generate equivalent atoms: #1, −*x* + 2, −*y* + 1, −*z*, (b) Coordination modes of the ligands found in **UCY-16**·6*n*DMF·*n*H_2_O.

The asymmetric unit of **UCY-16**·6*n*DMF·*n*H_2_O consists of six copper(II) cations, three hydroxide anions, five 5-aminoisophthalate ligands, as well as six DMF and one water lattice solvent molecules. The secondary building units (SBUs) of this compound are two tetranuclear clusters in which the four Cu^2+^ cations are held together through two monoatomic triply bridging hydroxides (μ_3_–OH^–^) giving rise to [Cu_4_(μ_3_–OH)_2_]^6+^ butterfly–like subunits. The peripheral ligation of one of these [Cu_4_(μ_3_–OH)_2_]^6+^ subunits (containing atoms Cu(1)–Cu(4) and O(1)/O(2)) is completed by seven carboxylate ligands and two terminal −NH_2_ groups from nine different 5-aminoisophthalate anions, whereas this of the second one (containing atoms Cu(5), Cu(6) and O(3)) by 6 carboxylate ligands and two terminal −NH_2_ groups from eight different 5-aminoisophthalate groups (Figure 1a).

The AIP ligands can be separated in two groups (Figure 1b): (a) ligands **B**, **C** and **E**, which connect five Cu^2+^ ions (i.e., μ_5_-ligands), using all their donor atoms; their carboxylates bridge two Cu^2+^ ions adopting the common *syn-syn* mode and the amino group acts as a terminal ligand and (b) ligands **A** and **D** which connect three Cu^2+^ ions (i.e., μ_3_-ligands) using their two carboxylate groups (the amino group remains unbound); one of their carboxylates bridges two metal ions in the common syn-syn mode whereas the other one is terminally ligated to Cu^2+^ ion either in a *syn* fashion (ligand **A**) or in an *anti*-fashion (ligand **D**) (Figure 1b). Charge balance considerations indicate that one of the aminoisophthalate ligands exhibits −1 charge; this may happen either because one of the terminally ligated carboxylate groups of ligands **A** or **D** is not deprotonated or because one of the unbound amino groups of the same ligands is protonated.

**Figure 2:**
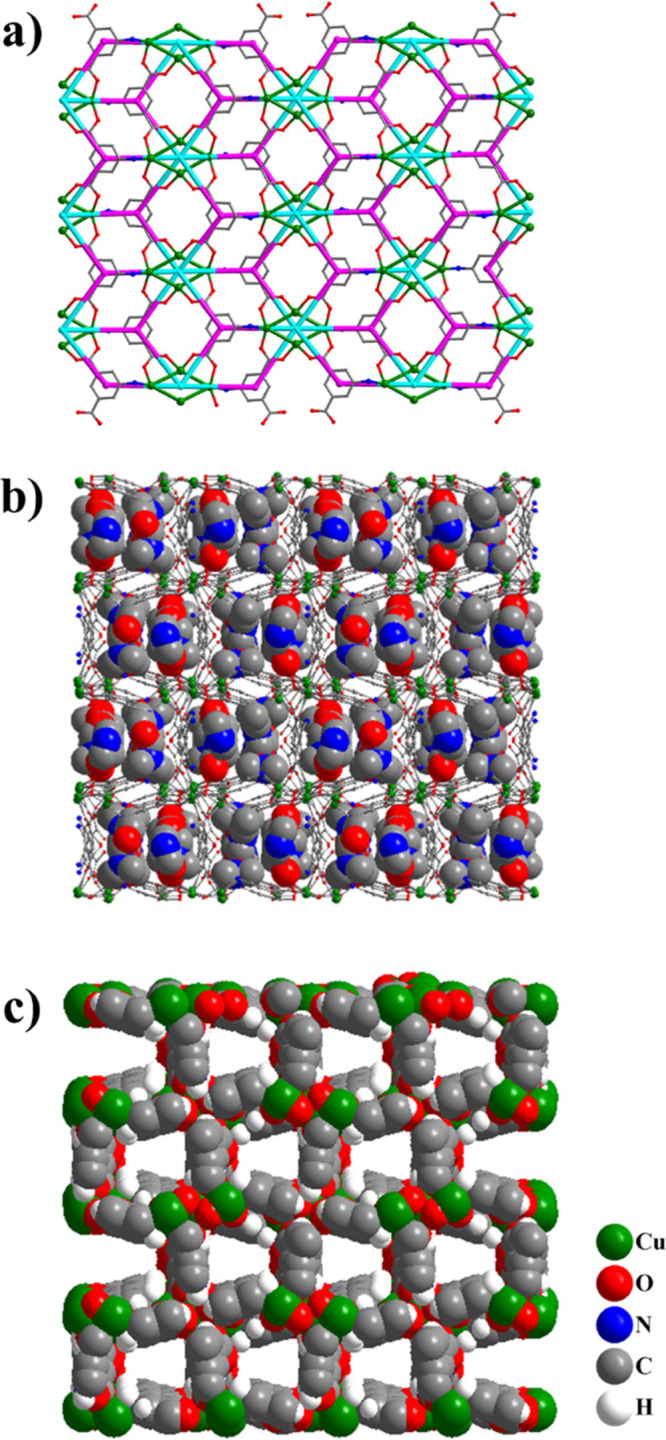
(a) Connectivity of the butterfly-like SBUs and B, C, and E ligands on the ac plane of the unit cell and the underlying **kgd** topology. Color code: butterfly centroid, sky blue; AIP aromatic ring centroid, pink. (b) Representation of the 3D framework of **UCY-16**·6*n*DMF·*n*H_2_O emphasizing the lattice solvent molecules (space-filling model) occupying the solvent accessible volume of **UCY-16**·6*n*DMF·*n*H_2_O (ball and stick model). (c) Space-filling representation of the framework of **UCY-16**·6*n*DMF·*n*H_2_O along the *c*-axis emphasizing the rectangular channels with an approximate diameter ∼6 Å.

The tetranuclear butterfly–like SBUs are located on a thick layer with an approximate thickness of 7 Å running parallel to the *ac* plane of the unit cell. The neighboring butterfly–like SBUs are connected on the *ac* plane through the AIP^2–^ ligands **B**, **C**, and **E**; each butterfly is connected to six ligands via four bridging carboxylates and two amino nitrogen atoms, being 6 connected nodes while the AIP^2–^ ligands are three connected nodes leading to the formation of a binodal kagome dual plane net (**kgd**, point symbol: {4^3^}_2_{4^6^.6^6^.8^3^}) (Figure 2a). The connection of the **kgd** dual plane nets is achieved by the linkers **A** and **D** resulting in the formation of a 3D framework. The structural description of **UCY-16**·6*n*DMF·*n*H_2_O, however, is getting complicated when ligands **A** and **D** are considered; those in addition to the different coordination modes of the two butterflies above and below the *ac* plane lead to a unique and complex topological network which is discussed in detail in the SI (Figures S1 and S2).

The framework pores accommodate guest solvent molecules (water and DMF) that are involved in hydrogen bonding interactions with the amine and the carboxylate groups of the AIP^2–^/HAIP^–^ ligands as well as the bridging μ_3_–OH^–^ groups of the SBUs (Figures 2b and S3). The solvent accessible volume was calculated by PLATON not considering the lattice solvent molecules and was found to be ∼6682 Å^3^, which corresponds to 48.5% of the unit cell volume. The 3D structure contains rectangular channels along the *c*-axis with an approximate diagonal of ∼6 Å as found by PLATON (considering the van der Waals radii of the atoms and excluding all solvents of the pores)^82^ (Figure 2c).

### Chemical and Thermal Stability

The stability of the compound was studied in various common organic solvents as well as in water. Suspensions of compound **UCY-16**·6*n*DMF·*n*H_2_O (50 mg) in each solvent (10 mL) were left under magnetic stirring for 3 days (concerning the stability studies in organic solvents) or 1 day (stability studies in water). The solid was collected by filtration and analyzed by pXRD. The resulting diffractograms showed that compound **UCY-16**·6*n*DMF·*n*H_2_O is stable in the organic solvents tested but decomposes in water (Figures S5 and S6).

The thermal stability of compound **UCY-16**·6*n*DMF·*n*H_2_O was studied by means of thermogravimetric analysis (20–600 °C) (Figure S7) and variable temperature (VT) pXRD (20–300 °C) measurements (Figure S8). The decomposition of **UCY-16**·6*n*DMF·*n*H_2_O is a multistep process which begins almost at room temperature. The first step is attributed to the removal of the lattice solvent molecules and is completed at ∼250 °C (calculated loss ≈25.6%; found ≈25%). The initial lattice solvent molecule loss is followed by the decomposition of the framework, which is completed at ∼450 °C (calculated loss ≈47.7%; found ≈48%). The residue at 600 °C corresponds to CuO (calculated residue ≈26.7%; found ≈27%). VT-pXRD measurements of the compound **UCY-16**·6*n*DMF·*n*H_2_O indicate that the framework collapses above 250 °C (Figure S8).

### SCSC Guest Solvent Exchange Transformations

**Figure 3:**
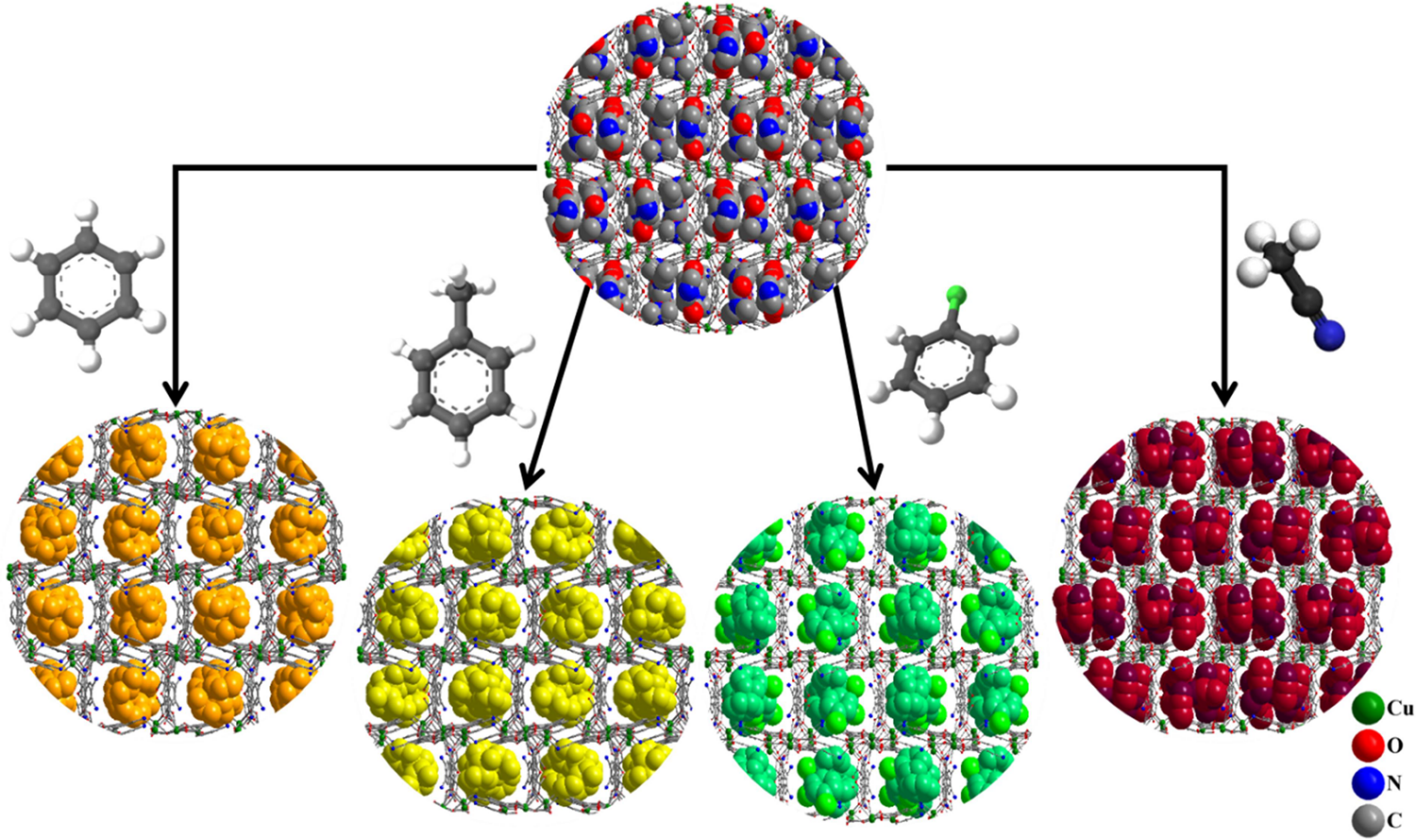
Representations of the 3D frameworks of **UCY-16**·6*n*DMF·*n*H_2_O and **UCY-16**/S (S = Bz, Tol, PhCl, and MeCN). The framework is represented in the ball and stick model, whereas the lattice solvent molecules are represented in the spacefill model. Color code: C gray; O red; N blue; Cu green; Bz orange; Tol yellow; PhCl green; MeCN burgundy.

Considering the relatively large solvent accessible volume, the suitable pore size for the insertion of solvent molecules (∼6 Å), the presence of various functionalities (i.e., −NH_2_, −OH^–^ and aromatic sites of the ligands), and the excellent quality of the single crystals of compound **UCY-16**·6*n*DMF·*n*H_2_O, it was decided to explore its SCSC transformation properties. In particular, the reactions of **UCY-16**·6*n*DMF·*n*H_2_O with selected aromatic and aliphatic solvent molecules S (S = Bz, Tol, PhCl, and MeCN) were investigated since their kinetic diameters are close to the pore size of **UCY-16**·6*n*DMF·*n*H_2_O.^86^ Heterogeneous SCSC transformation reactions of single crystals of **UCY-16**·6*n*DMF·*n*H_2_O with the aromatic organic solvents were conducted under autogenous pressure at 100 °C, whereas the reaction with MeCN took place at room temperature (Figures 3 and S9–S12).

These reactions resulted in single crystals which were macroscopically very similar in size and shape to those of the pristine compound, and X-ray structural determination of the exchanged analogues revealed that compounds **UCY-16**/S (S = Bz, Tol, PhCl, and MeCN) have very similar structures to that of the pristine **UCY-16**·6*n*DMF·*n*H_2_O (Figure 3). Note that for compounds **UCY-16**/S (S = Bz, Tol, PhCl, and MeCN), several single crystals were examined and proven to have identical unit cell parameters. These exchange reactions led to the insertion of six MeCN, three Bz or PhCl, and two Tol solvent molecules giving rise to the formation of compounds **UCY-16**·3Bz·DMF·2H_2_O, **UCY-16**·3Tol·DMF·2H_2_O, **UCY-16**·3PhCl·H_2_O, and **UCY-16**·6MeCN·2H_2_O. Note that in the case of **UCY-16**/Tol, a third Tol molecule was also located during the structure solution which was severely disordered and could not be modeled properly, and for this reason, it was removed using the SQUEEZE routine of the PLATON package.^82^ The Bz, Tol, and PhCl molecules of compounds **UCY-16**/S are in close proximity with AIP^2–^/HAIP^–^ ligands displaying edge to face π···π stacking (as well as CH_3_···π interactions in the case of Tol) interactions (Figures S9–S11). On the other hand, MeCN was expected to behave differently than the aromatic organic molecules in these SCSC reactions because it exhibits a significant capability to be involved in hydrogen bonding interactions. This was also proven experimentally since the structure of **UCY-16**/MeCN features several MeCN and H_2_O molecules interacting through relatively strong H-bonds with the framework (2.81–3.08 Å) (Figure S12). In particular, the MeCN molecules are hydrogen bonded with the μ_3_-ΟΗ^–^ bridges as well as with the nitrogen atoms of the AIP^2–^/HAIP^–^ ligands. In addition, the two lattice water molecules of **UCY-16**/MeCN are also involved in hydrogen bonding interactions (Figure S12).

A close inspection of the unit cell parameters of the pristine compound **UCY-16**·6*n*DMF·*n*H_2_O and the exchanged analogues **UCY-16**/S (S = Bz, Tol, PhCl, and MeCN) revealed that the unit cell of the framework slightly contracts upon insertion of the solvent molecules into the framework. The main modification of the unit cell parameters in the exchanged analogues is a decrease of the *b*-axis dimension of the unit cell by up to ∼3%, which is responsible for the overall decrease of the unit cell volume. The contraction of the framework upon the insertion of solvent molecules is also reflected on the solvent accessible volume of the exchanged analogues which are (∼6–11%) smaller than that of the pristine compound. (Table S2) Other significant structural modifications also appear in the structures of the exchanged analogues including the decrease of selected bond lengths compared to the corresponding ones of the pristine compound, mainly along the b–axis. Specifically, the Cu–O distances of the monodentate carboxylate groups of ligands **A** (Cu4–O22) and **D** (Cu2–O4) in **UCY-16**/S display average values of 2.04 and 2.55 Å, which are shorter than the corresponding ones of the pristine compound which are 2.21 and 2.68 Å, respectively.

pXRD studies and IR spectra of the exchanged analogues confirmed that the crystallinity and structural integrity of compounds **UCY-16**/S (S = Bz, Tol, PhCl, MeCN) is retained after the SCSC reactions (Figures S13 and S14). TG analysis revealed that the decomposition of **UCY-16**/S (S = Bz, Tol, PhCl, MeCN) is completed in two steps (Figure S15). The first one is attributed to the release of lattice solvent molecules and is completed at 255–275 °C, whereas the second one is completed at 435–465 °C and is attributed to the combustion of AIP^2–^/HAIP^–^ ligands. Finally, the residue at 600 °C corresponds to CuO. (Table S3) The SCSC transformations discussed so far included insertion in the framework of **UCY-16** of guest aromatic molecules and a polar aprotic solvent (i.e., MeCN) which illustrated the stability of **UCY-16**·6*n*DMF·*n*H_2_O in different organic solvents as well as the ability to sorb selected organic molecules.

### SCSC Bridging Ligand Exchange Transformations

**Figure 4:**
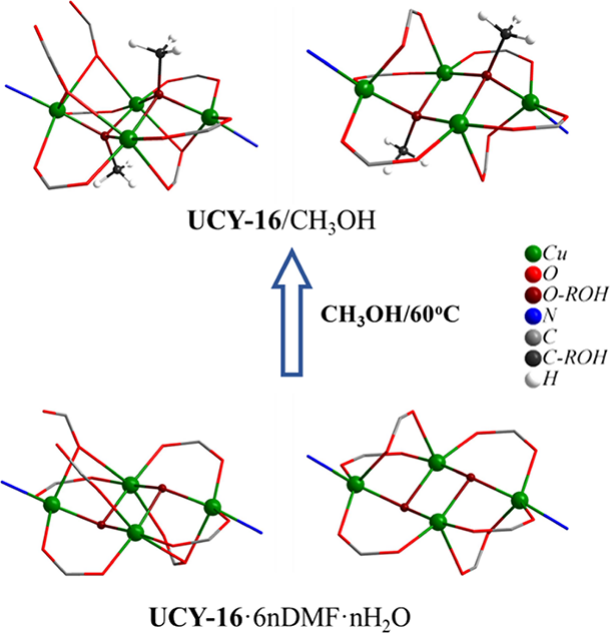
Representations of the SBUs of **UCY-16**·6*n*DMF·*n*H_2_O. and **UCY-16**/CH_3_OH emphasizing on the exchange of the monoatomic μ_3_–OH^–^ bridges by μ_3_-ΟMe^–^ groups. The metal ions and μ_3_- bridges are represented in the ball and stick model, whereas the remaining atoms of the SBUs are in the wireframe model.

Further study of the SCSC properties of **UCY-16**·6*n*DMF·*n*H_2_O included the investigation of reactions with polar protic solvents such as primary alcohols. Indeed, single crystals of **UCY-16**·6*n*DMF·*n*H_2_O were employed in heterogeneous reactions with primary alcohols at elevated temperatures (60–150 °C). These crystals remained almost unchanged in size and shape and were of sufficient quality for crystallographic analysis. Specifically, the reaction of **UCY-16**·6*n*DMF·*n*H_2_O with MeOH at 60 °C revealed that not only the DMF lattice solvent molecules were exchanged by MeOH but also the μ_3_-ΟΗ^–^ monoatomic bridges of the butterfly-like [Cu_4_(μ_3_–OH)_2_]^6+^ structural core of the SBUs were replaced by bridging μ_3_-ΟMe^–^ groups (Figure 4).

This type of transformation, which involves the SCSC exchange of a bridging ligand that contributes to the formation of the SBU and not necessarily to the formation of the framework is quite rare in MOF chemistry. There are a series of SCSC ligand exchange/installation transformations reported including the replacement of terminal solvent molecules by various organic ligands,^54−56^ or polytopic ligands by other ones,^60,87,88^ or installation of bridging polytopic ligands.^60,68,69^ However, to the best of our knowledge, there are only two examples involving the exchange of a monoatomic bridging ligand of the SBU of a MOF, an In^3+^ MOF where a monoatomic μ–ΟΗ^–^ bridge is exchanged by a μ–ΟMe^–^ group^89^ and a Cu^2+^ MOF where a bridging solvent molecule (μ-H_2_O) is replaced by other ones (μ-DMSO or μ-MeOH).^90^

**Figure 5:**
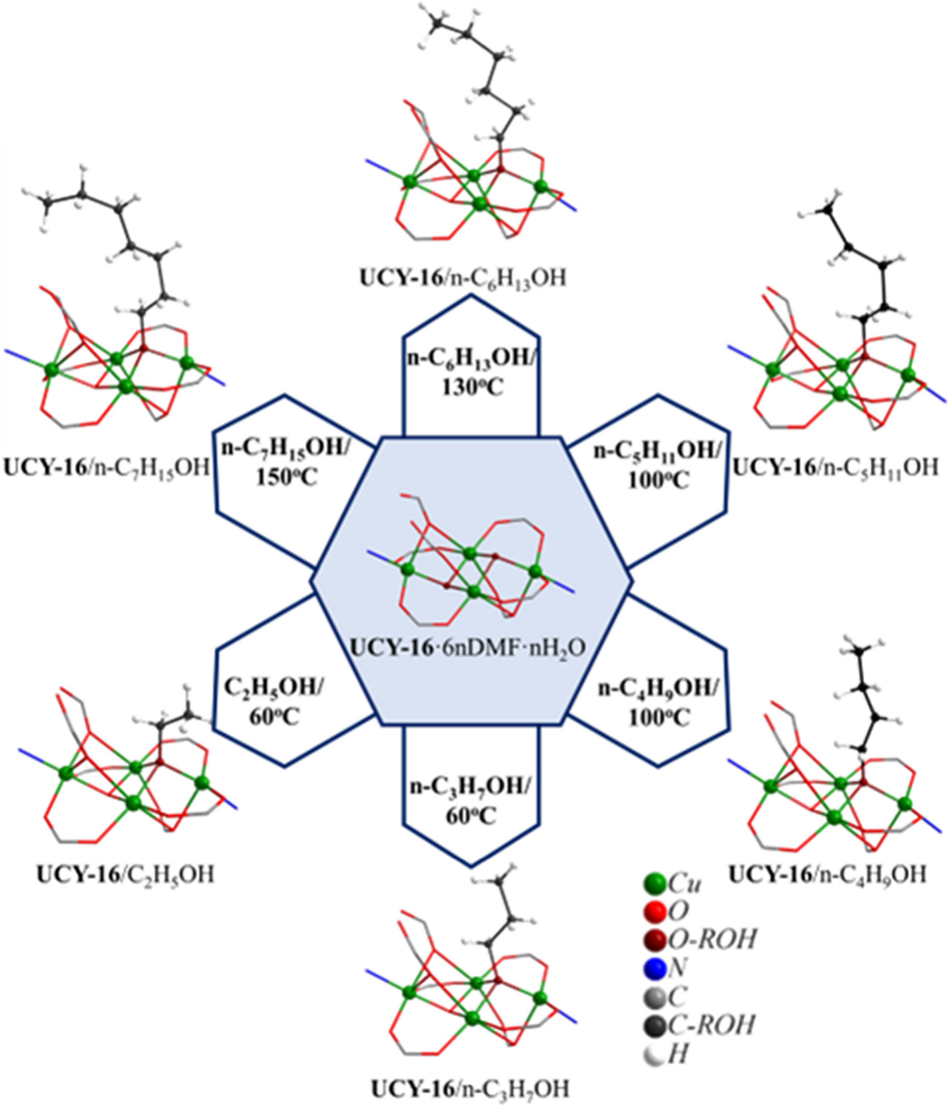
Representations of the SBUs of **UCY-16**·6*n*DMF·*n*H_2_O and **UCY-16**/*n*-C_*x*_H_2*x*+1_OH (*x* = 2–7) emphasizing on the exchange of a monoatomic μ_3_–OH^–^ bridge by the corresponding μ_3_-OR^–^ group. The μ_3_- bridges involved in the exchange reactions and the metal ions are represented in the ball and stick model, whereas the remaining atoms of the SBUs are represented in the wireframe model.

Similar reactions of **UCY-16**·6*n*DMF·*n*H_2_O with additional primary alcohols *n*-C_*x*_H_2*x*+1_OH (*x* = 2–7) were also investigated and resulted in the formation of compounds **UCY-16**/*n*-C_*x*_H_2*x*+1_OH (*x* = 2–7). In all these compounds, only one μ_3_-ΟΗ^–^ monoatomic bridge of the butterfly-like SBUs was replaced by a bridging μ_3_-OR^–^ group in contrast to the situation in **UCY-16**/CH_3_OH (Figure 5). Specifically, the μ_3_–OH^–^ bridge that is replaced by a μ_3_-OR^–^ group in the exchanged analogues **UCY-16**/*n*-C_*x*_H_2*x*+1_OH (*x* = 2–7) is the one connecting the Cu^2+^ ions Cu(1)–Cu(3). The replacement of only this group could be possibly attributed to steric hindrance in the other positions since the monoatomic bridges that connect Cu(2)/Cu(3)/Cu(4) and Cu(5)(two symmetry–related metal ions)/Cu(6) ions contain above and in close proximity AIP^–^/HAIP^–^ ligands. Moreover, the coordination modes of the AIP^2–^/HAIP^–^ ligands of the 3D framework of **UCY-16**/*n*-C_*x*_H_2*x*+1_OH (*x* = 2–7) remain almost identical upon the SCSC exchange reactions. Notably, the decrease of the bond lengths of the monodentate carboxylate groups of ligands **A** and **D** is also observed (the distance Cu(4)–O(22) is decreased from 2.21 to2.07 Å and for the Cu(2)–O(4) one from 2.68 to 2.54 Å) as discussed for compounds **UCY-16**/S (S = Bz, Tol, PhCl, and MeCN). In addition, the contraction of unit cell dimensions (mainly of b-axes) and unit cell volume is also observed in the family of **UCY-16**/*n*-C_*x*_H_2*x*+1_OH (*x* = 1–7) exchanged analogues. A close examination of the crystal structures of **UCY-16**/*n*-C_*x*_H_2*x*+1_OH (*x* = 1–7) revealed the existence of different types of interactions between the lattice solvent molecules and the framework of **UCY-16** in the various exchanged analogues. Specifically, the alcohols *n*-C_*x*_H_2*x*+1_OH (*x* = 1–5) of the corresponding exchanged MOFs interact mainly through hydrogen bonding interactions (Figures S16–S20), whereas the longer chain alcohols *n*-C_*x*_H_2*x*+1_OH (*x* = 6–7) are not involved in significant H-bonding interactions (Figures S21 and S22). In fact, the existence of longer chain alcohols such as 1-pentanol, 1-hexanol, and 1-heptanol in the structures of **UCY-16**/*n*-C_*x*_H_2*x*+1_OH (*x* = 5–7) (Figures S20–S22) is another uncommon structural feature of these compounds. Interestingly, the analogue **UCY-16**/*n*-C_7_H_15_OH represents the first example where lattice 1-heptanol molecules are observed in the pores of a MOF, whereas 1-pentanol and 1-hexanol have appeared only once previously in the structure of MOFs.^91,92^

pXRD and IR studies of the exchanged analogues **UCY-16**/*n*-C_*x*_H_2*x*+1_OH (*x* = 1–7) confirmed that they retain their crystallinity and structural integrity after the SCSC reactions. (Figures S23 and S24) Stability studies of the exchanged analogues containing longer–chain alcohols **UCY-16**/*n*-C_*x*_H_2*x*+1_OH (*x* = 6–7) revealed an increased stability in water compared to the one of the pristine MOF (Figure S25). However, there is a loss of crystallinity of the **UCY-16**/*n*-C_6_H_13_OH analogue upon treatment with water, something that is also observed for **UCY-16**/*n*-C_7_H_15_OH but to a significantly smaller extent.

Further efforts to enhance the hydrophobicity of **UCY-16** included investigation of postsynthesis modification reactions of **UCY-16**·6*n*DMF·*n*H_2_O with longer - chain primary alcohols. Thus, heterogeneous reactions of the pristine MOF **UCY-16**·6*n*DMF·*n*H_2_O with the primary alcohols *n*-C_*x*_H_2*x*+1_OH (*x* = 8–10, 12, 14, and 16) were carried out at elevated temperatures and resulted in the isolation of microcrystalline powders (Figures S23 and S24). Although we were unable to obtain the crystal structures of the **UCY-16**/*n*-C_*x*_H_2*x*+1_OH (*x* = 8–10, 12, 14, and 16) exchanged analogues, it was confirmed from the powder diffractograms that they retain their crystallinity and structural integrity (Figure S23). In addition, infrared spectra obtained for these exchanged analogues are in line with this of the pristine material and contain the expected bands in the range 2700–3000 cm^–1^ due to the presence of the C–H bonds of the long–chain fatty alcohols (Figure S24). From this characterization, it is clear that the long–chain fatty alcohols are present in the **UCY-16**/*n*-C_*x*_H_2*x*+1_OH (*x* = 8–10, 12, 14, and 16) exchanged analogues; however, it is impossible to conclude whether they appear in the structure of the SBU (replacing one or more μ_3_–OH^–^ anions) or in the lattice of the MOF or even in the outer surface of the materials. Stability studies of the exchanged analogues **UCY-16**/*n*-C_*x*_H_2*x*+1_OH (*x* = 8–10, 12, 14 and 16) indicated that their crystallinity is retained upon treatment in water for 24 h (Figure S26). The thermal stability of the exchanged compounds **UCY-16**/*n*-C_*x*_H_2*x*+1_OH (*x* = 1–10, 12, 14, and 16) was studied by means of TGA which revealed that their decomposition is completed in two steps (Figures S27 and S28). The first step is attributed to the release of lattice alcohol molecules and residual water or DMF lattice solvents and is completed at 250–270 °C, whereas the second step is completed at 500 °C and is attributed to the combustion of AIP^2–^/HAIP^–^ ligands as well as the bridging μ_3_-OC_*x*_H_2*x*+1_^–^ (*x* = 1–10, 12, 14 and 16) groups. Finally, the residue at 600 °C corresponds to CuO (Table S4).

### Tuning the Hydrophobicity–Application

**Figure 6:**
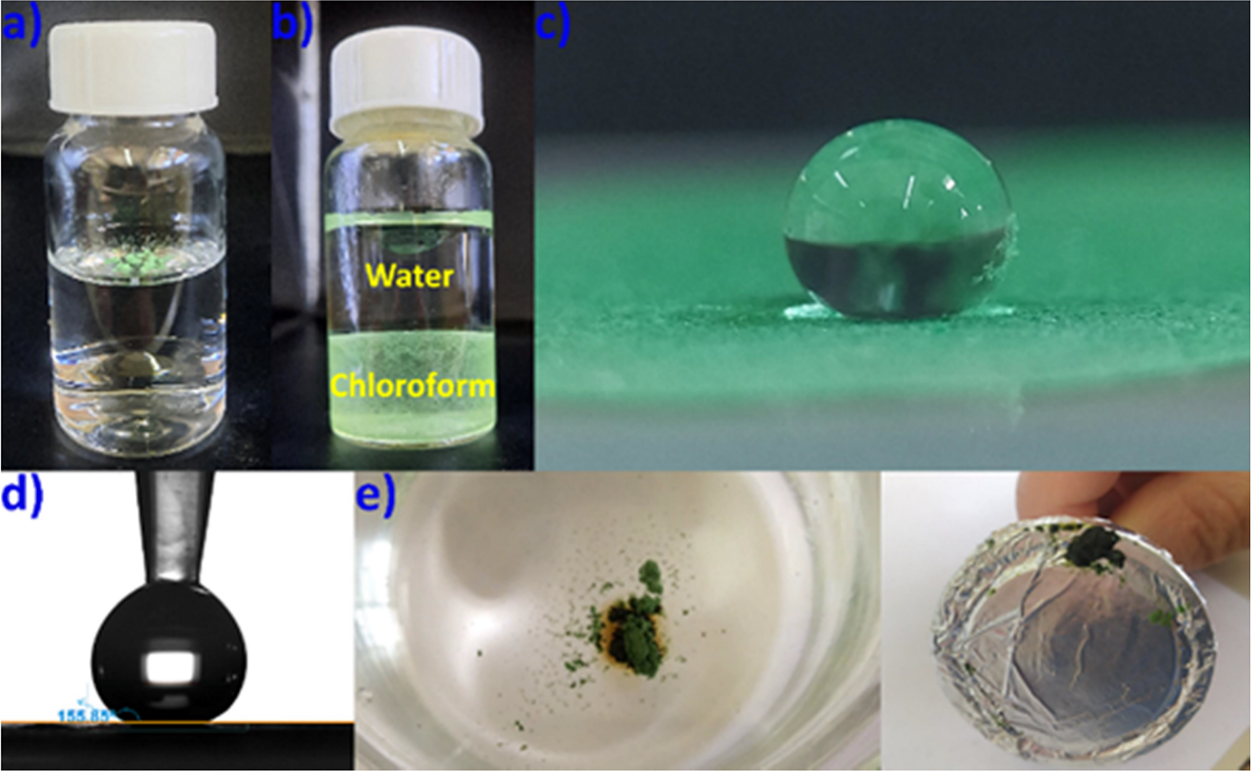
Images of microcrystalline powder of **UCY-16**/*n*-C_16_H_33_OH (a) floating on the water surface and (b) dispersing in chloroform. (c) Distilled water droplet (10 μL) on a thin film of **UCY-16**/*n-*C_16_H_33_OH. (d) Digital photograph of the water contact angle for **UCY-16**/*n*-C_16_H_33_OH measured by a contact angle goniometer. (e) Crude oil removal from the water surface using the magnetic composite **UCY-16**/*n*-C_16_H_33_OH/Fe_3_O_4_.

SCSC transformation and PSM exchange reactions of **UCY-16**·6*n*DMF·*n*H_2_O with long-chain fatty alcohols were proven to be successful approaches for the tuning of its wetting properties. The use of organic linkers with extended aliphatic or fluorinated functional groups in MOF synthesis typically results in materials with low surface energy that interact weakly with water. Consequently, such materials present hydrophobic or even superhydrophobic properties.^93−95^ The series of MOFs discussed and in particular the microcrystalline powder of the analogues with long-chain alcohols **UCY-16**/*n*-C_*x*_H_2*x*+1_OH·S′ (*x* = 6, 8, 9, 10, 12, 14, 16) were floating on the water surface which was the first evidence of their hydrophobic/superhydrophobic properties (Figures 6a ad S29). In addition, the hydrophobic materials easily disperse in nonpolar solvents (such as CHCl_3_), while being immiscible with water (Figure 6b). Water contact angle (WCA) studies indicated that the MOFs with alkyl chains C_*x*_H_2*x*+1_ (*x* = 6, 8–10, 12, and 14) were hydrophobic with WCA values >140 ± 5° (Figures 6c, S30, and S31), while **UCY-16**/*n*-C_16_H_33_OH was superhydrophobic with a WCA of 156 ± 5° (Figures 6d, S30, and S31). The hydrophobic/superhydrophobic MOFs were converted into a form that could be easily retrieved after an oil–water separation procedure by synthesizing a magnetic superhydrophobic MOF composite. This was prepared by mixing **UCY-16**/*n*-C_16_H_33_OH and Fe_3_O_4_ (mass ratio of 3:1) in acetone at ambient temperature. The magnetic superhydrophobic MOF composite **UCY-16**/*n*-C_16_H_33_OH/Fe_3_O_4_ was then investigated for its capability to remove crude oil from water. As shown in Figures 6e and S32, and Video S1, the MOF-based magnetic composite was able to quickly sorb oil from the surface of a water sample, and the oil-laden material could be easily recovered using a magnet.

## Conclusions

SCSC exchange reactions have gained significant attention in the past few years as a versatile method to achieve targeted structural alterations and fine tune the properties of functional MOFs. A new Cu^2+^ 3-dimensional MOF with the formula [Cu_6_(μ_3_-ΟΗ)_3_(ΑΙΡ)_4_(ΗΑΙΡ)]_*n*_·6*n*DMF·*n*H_2_O - **UCY-16**·6*n*DMF·*n*H_2_O is reported exhibiting interesting SCSC transformation properties. The pristine material **UCY-16**·6*n*DMF·*n*H_2_O was isolated in high yield using low-cost starting materials and is based on two different SBUs displaying the same [Cu_4_(μ_3_–OH)_2_]^6+^ butterfly-like structural cores which are connected through five ΑΙΡ^2–^/ΗΑΙΡ^–^ ligands. Thermal, chemical, and hydrolytic stability studies of **UCY-16**·6*n*DMF·*n*H_2_O indicated that it is thermally stable up to 250 °C and remains intact in most organic solvents but not in water. This compound displayed a significant capability to exchange in a SCSC fashion its lattice solvent molecules as well as coordinated groups as confirmed by the isolation of 11 new exchanged products. Some of the obtained modifications exhibited significant elements of novelty including the replacement of the monoatomic bridging μ_3_–OH^–^ anion of the [Cu_4_(μ_3_–OH)_2_]^6+^ butterfly-like core of the SBU which has not been shown previously. In addition, the SCSC insertion of long–chain alcohols (*n*-C_*x*_H_2*x*+1_OH; *x* = 5–7) in the structure of a MOF is another uncommon structural feature and reactivity feature. Further investigation of exchange reactions of the pristine MOF with long-chain fatty alcohols allowed the preparation of a series of postsynthetically modified analogues **UCY-16**/*n*-C_*x*_H_2*x*+1_OH (*x* = 8–10, 12, 14, and 16), which are immiscible with water and display significant hydrolytic stability. Water contact angle measurements of **UCY-16**/*n*-C_*x*_H_2*x*+1_OH·S′ (*x* = 6, 8–10, 12, 14, and 16) indicated their hydrophobic/superhydrophobic nature. The magnetic superhydrophobic MOF composite **UCY-16**/*n*-C_16_H_33_OH/Fe_3_O_4_ was synthesized and demonstrated to be very efficient in removing crude oil from water under static conditions. Furthermore, it could be readily recovered, along with the organic pollutant, using an external magnet. This result indicated that the series of functionalized **UCY-16**/*n*-C_*x*_H_2*x*+1_OH·S′ analogues might be promising for multiple applications related to oil–water separation processes, including wastewater purification in crude oil production and oil spill cleanup. Thus, a detailed investigation of the SCSC transformation properties of **UCY-16**·6*n*DMF·*n*H_2_O allowed the development of a facile method for the fine-tuning of the hydrophobicity of MOFs. Overall, this work demonstrates an unprecedented SCSC transformation method for the fine-tuning of the hydrophobicity of MOFs and its use for the development of materials capable of removing hydrophobic pollutants from aqueous media.

## Supporting Information Available

Crystallography details, pXRD, IR, TGA graphs and analysis, structural figures, and physical measurements and characterization (PDF)
Crude oil removal by **UCY-16**/*n*-C_16_H_33_OH/Fe_3_O_4_ (mass ratio of 3:1) and recovery of the oil-laden material using an external magnet (MP4)
