# Supplementary material for: Fine Tuning the Hydrophobicity of a New Three-Dimensional Cu2+ MOF through Single Crystal Coordinating Ligand Exchange Transformations
Source: Inorg Chem. 2024 Feb 9;63(8):3824–34. doi: 10.1021/acs.inorgchem.3c04060 (PMC10900299; doi:10.1021/acs.inorgchem.3c04060)
Supplement: Supplementary file 1 — ic3c04060_si_001.pdf [file ic3c04060_si_001.pdf]

Supporting Information (SI)

Fine Tuning the Hydrophobicity of a New 3-Dimensional Cu^{2+} MOF Through Single - Crystal Coordinating Ligand Exchange Transformations

Nikos Panagiotou[a], Dimitrios A. Evangelou[b], Manolis J. Manos[b], John C. Plakatouras[b], Anastasios J. Tasiopoulos*[,a]

[a] *Department of Chemistry, University of Cyprus, 1678 Nicosia, Cyprus.*

[b] *Department of Chemistry, University of Ioannina, 45110 Ioannina, Greece*

Email: atasio@ucy.ac.cy

Table of Contents

Single Crystal X-ray Crystallography Tables S3

Extended structural/topological description of **UCY-16**·6nDMF·nH_2O S6

Physical Measurements/Characterization of **UCY-16**·6nDMF·nH_2O S9

Structural figures of **UCY-16**/S (S = Bz, Tol, PhCl and MeCN) S12

Physical Measurements/Characterization of compounds **UCY-16**/S (S = Bz, Tol, PhCl and MeCN) S15

Structural figures of (**UCY-16**/n-$C_xH_{2x+1}OH$)·S′ (x = 1-7; S′ = lattice solvents) S17

Physical Measurements/Characterization of compounds of **(UCY-16**/n-$C_xH_{2x+1}OH$)·S′ (x = 1-10, 12, 14 and 16; S′ = lattice solvents) S24

Single Crystal X-ray Crystallography Tables

Table S1: Selected Crystal Data for **UCY-16**·6nDMF·nH$_2$O and **UCY-16**/S (S = Benzene (Bz), Toluene (Tol), Chlorobenzene (PhCl), MeCN)

Compound	**UCY-16**·6nDMF·nH$_2$O	**UCY-16**/Bz	**UCY-16**/Tol	**UCY-16**/PhCl	**UCY-16**/MeCN
Empirical formula	$C_{58}H_{72}N_{11}O_{30}Cu_6$	$C_{61}H_{50}N_6O_{26}Cu_6$	$C_{57}H_{48}N_6O_{26}Cu_6$	$C_{58}H_{40}N_5O_{25}Cl_3Cu_6$	$C_{52}H_{43}N_{11}O_{25}Cu_6$
Formula weight	1784.50	1664.31	1614.25	1694.54	1603.21
Temperature/K	100 (1)	100 (1)	100 (1)	100 (1)	100 (1)
Crystal system	Orthorhombic	Orthorhombic	Orthorhombic	Orthorhombic	Orthorhombic
Space group	Pbca	Pbca	Pbca	Pbca	Pbca
a/Å	21.6852(6)	21.6223(3)	21.6491(2)	21.6946(3)	21.5476(3)
b/Å	19.9666(4)	19.3945(3)	19.6086(3)	19.5287(4)	19.2990(3)
c/Å	31.8129(6)	31.7827(4)	31.7929(3)	31.8271(4)	31.8441(4)
α = β = γ /°	90	90	90	90	90
Volume/Å^3	13774.3(5)	13328.2(3)	13496.4(3)	13484.1(4)	13242.3(3)
Z	8	8	8	8	8
ρ_{calc}g/cm^3	1.721	1.659	1.589	1.669	1.608
μ/mm^{-1}	2.852	2.838	2.781	3.871	2.841
F(000)	7288	6720	6512	6784	6448
Radiation	Cu Kα (λ = 1.54184)	Cu Kα (λ = 1.54184)	Cu Kα (λ = 1.54184)	Cu Kα (λ = 1.54184)	Cu Kα (λ = 1.54184)
Reflections collected	50904	46090	50815	48200	32215
Independent reflections	12249 [R_{int} = 0.0401, R_{sigma} = 0.0316]	11860 [R_{int} = 0.0405, R_{sigma} = 0.0281]	12025 [R_{int} = 0.0320, R_{sigma} = 0.0236]	12011 [R_{int} = 0.0412, R_{sigma} = 0.0336]	11771 [R_{int} = 0.0224, R_{sigma} = 0.0242]
Data/restraints/parameters	12249/0/949	11860/0/856	12025/114/832	12010/232/890	11771/45/853
Goodness-of-fit on F^2	1.059	1.035	1.04	1.087	1.044
Final R indexes [I>=2σ (I)]	R_1 = 0.0434, wR_2 = 0.1208	R_1 = 0.0658, wR_2 = 0.1832	R_1 = 0.0680, wR_2 = 0.1923	R_1 = 0.0807, wR_2 = 0.2398	R_1 = 0.0803, wR_2 = 0.2232
Final R indexes [all data]	R_1 = 0.0514, wR_2 = 0.1269	R_1 = 0.0737, wR_2 = 0.1917	R_1 = 0.0753, wR_2 = 0.2006	R_1 = 0.0983, wR_2 = 0.2574	R_1 = 0.0867, wR_2 = 0.2286

[a]$R = \Sigma||F_o|-|F_c|| / \Sigma|F_o|$, $wR = \{\Sigma[w(|F_o|^2 - |F_c|^2)^2] / \Sigma[w(|F_o|^4)]\}^{1/2}$ and [b]$w=1/[\sigma^2(F_o^2)+(mP)^2+nP]$ where $P=(Fo^2+2Fc^2)/3$ and m and n are constants

Table S1(continued): Selected Crystal Data for (**UCY-16**/n-$C_xH_{2x+1}OH$)·S′ (x = 1, 2, 3, 4, 5, 6, 7)

Compound	**UCY-16**/CH_3OH	**UCY-16**/C_2H_5OH	**UCY-16**/n-C_3H_7OH	**UCY-16**/n-C_4H_9OH	**UCY-16**/n-$C_5H_{11}OH$	**UCY-16**/n-$C_6H_{13}OH$	**UCY-16**/n-$C_7H_{15}OH$
Empirical formula	$C_{93}H_{89}N_{10}O_{61}Cu_{12}$	$C_{54}H_{60}N_5O_{31}Cu_6$	$C_{58}H_{67}N_5O_{28}Cu_6$	$C_{47.5}H_{42}N_{5.5}O_{24.5}Cu_6$	$C_{60}H_{69}N_5O_{26}Cu_6$	$C_{52}H_{51}N_5O_{24}Cu_6$	$C_{54}H_{55}N_5O_{24}Cu_6$
Formula weight	3085.22	1656.31	1663.4	1463.1	1657.44	1511.22	1539.27
Temperature/K	100 (1)	100 (1)	100 (1)	102.2 (2)	100 (1)	100 (1)	100 (1)
Crystal system	Orthorhombic	Orthorhombic	Orthorhombic	orthorhombic	Orthorhombic	Orthorhombic	Orthorhombic
Space group	Pcab	Pbca	Pbca	Pbca	Pbca	Pbca	Pbca
a/Å	31.774(5)	21.5536(3)	21.6280(5)	21.6666(3)	21.6532(4)	21.6882(4)	21.7273(4)
b/Å	19.485(5)	19.5236(3)	19.5122(4)	19.6612(3)	19.4801(5)	19.6805(4)	19.5734(3)
c/Å	21.451(5)	31.8040(4)	31.8170(7)	31.8338(3)	31.8076(5)	31.8245(4)	31.8114(5)
α = β = γ/°	90	90	90	90	90	90	90
Volume/Å^3	13281(5)	13383.2(3)	13427.1(5)	13560.9(3)	13416.7(5)	13583.8(4)	13528.7(4)
Z	4	8	8	8	8	8	8
ρ_{calc} g/cm^3	1.543	1.644	1.646	1.433	1.641	1.478	1.511
μ/mm^{-1}	2.841	2.869	2.827	2.685	2.804	2.691	2.713
F(000)	6212	6728	6784	5884	6768	6112	6240
Radiation	Cu Kα (λ = 1.54184)	Cu Kα (λ = 1.54184)	Cu Kα (λ = 1.54184)	Cu Kα (λ = 1.54184)	Cu Kα (λ = 1.54184)	Cu Kα (λ = 1.54184)	Cu Kα (λ = 1.54184)
Reflections collected	49109	46864	48238	30976	30640	52617	83506
Independent reflections	11857 [R_{int} = 0.0588, R_{sigma} = 0.0446]	11917 [R_{int} = 0.0344, R_{sigma} = 0.0281]	11956 [R_{int} = 0.0604, R_{sigma} = 0.0497]	12071 [R_{int} = 0.0297, R_{sigma} = 0.0360]	11935 [R_{int} = 0.0444, R_{sigma} = 0.0465]	12095 [R_{int} = 0.0564, R_{sigma} = 0.0408]	12052 [R_{int} = 0.0676, R_{sigma} = 0.0310]
Data/restraints/parameters	11857/38/808	11917/84/866	11956/654/874	12071/137/796	11935/192/874	12095/260/784	12052/134/802
Goodness-of-fit on F^2	1.333	1.092	1.163	1.07	1.031	1.103	1.061
Final R indexes [I>=2σ (I)]	R_1 = 0.0997, wR_2 = 0.3096	R_1 = 0.0774, wR_2 = 0.1941	R_1 = 0.0970, wR_2 = 0.2648	R_1 = 0.0566, wR_2 = 0.1639	R_1 = 0.0761, wR_2 = 0.2208	R_1 = 0.0891, wR_2 = 0.2513	R_1 = 0.0781, wR_2 = 0.2282
Final R indexes [all data]	R_1 = 0.1160, wR_2 = 0.3301	R_1 = 0.0869, wR_2 = 0.1989	R_1 = 0.1159, wR_2 = 0.2976	R_1 = 0.0701, wR_2 = 0.1724	R_1 = 0.0907, wR_2 = 0.2369	R_1 = 0.1082, wR_2 = 0.2706	R_1 = 0.0857, wR_2 = 0.2377

$^aR = \Sigma||F_o|-|F_c|| / \Sigma|F_o|$, $wR = \{\Sigma[w(|F_o|^2 - |F_c|^2)^2] / \Sigma[w(|F_o|^4)]\}^{1/2}$ and $^bw=1/[\sigma^2(F_o^2)+(mP)^2+nP]$ where $P=(Fo^2+2Fc^2)/3$ and m and n are constants

Table S2: Selected Crystal/Structural Data for **UCY-16**·6nDMF·nH_2O and **UCY-16**/S (S = Bz, Tol, PhCl, MeCN) (**UCY-16**/n-$C_xH_{2x+1}OH$)·S′ (x = 1, 2, 3, 4, 5, 6, 7)

Compound	a(Å)	b(Å)	c(Å)	V(Å^3)	SAV(Å^3)	SAV (%)	Space Group
**UCY-16**·6nDMF·nH_2O	21.69	19.97	31.81	13774	6682	48.5	Pbca
**UCY-16**/Bz	21.62	19.40	31.78	13328	6272	47.1	Pbca
**UCY-16**/Tol	21.65	19.61	31.79	13496	6179	45.8	Pbca
**UCY-16**/PhCl	21.70	19.53	31.83	13484	6454	47.9	Pbca
**UCY-16**/MeCN	21.55	19.30	31.84	13242	5946	44.9	Pbca
**UCY-16**/CH_3OH[a]	21.45	19.49	31.77	13281	5389	40.6	Pcab
**UCY-16**/C_2H_5OH	21.55	19.52	31.80	13383	5731	42.8	Pbca
**UCY-16**/n-C_3H_7OH	21.63	19.51	31.82	13427	5546	41.3	Pbca
**UCY-16**/n-C_4H_9OH	21.67	19.66	31.83	13561	5875	43.3	Pbca
**UCY-16**/n-$C_5H_{11}OH$	21.65	19.48	31.81	13417	5160	38.5	Pbca
**UCY-16**/n-$C_6H_{13}OH$	21.69	19.68	31.82	13584	5329	39.2	Pbca
**UCY-16**/n-$C_7H_{15}OH$	21.73	19.57	31.81	13529	5016	37.1	Pbca

[a]**UCY-16**/CH_3OH crystallizes in the Pcba space group thus the order of indices was switched to match the other exchanged analogues crystallizing in the Pbca space group.

Extended structural/topological description of **UCY-16**·6nDMF·nH_2O

The structural description of **UCY-16**·6nDMF·nH_2O (the asymmetric unit is shown in Fig. S1a) is getting complicated when ligands **A** and **D** are taken into account; those in addition to the different coordination behavior of the two butterfly – like secondary building units (SBUs) above and below *ac* plane lead to a unique topological network. Although the coordination spheres of the Cu^{2+} ions in the two butterflies are very similar [trigonal bipyramidal for the outer atoms Cu(1), Cu(4) and Cu(6) and Jan-Teller distorted square pyramidal for the inner atoms Cu(2), Cu(3) and Cu(5)] their connectivity is different due to symmetry. Butterfly Cu(1) – Cu(4) is connected to three (one **A** and two **D**) AIP ligands above and below the *ac* plane, while butterfly Cu(5) – Cu(6) is connected only to two inversion related **A** ligands. (Figure S1b)

a)

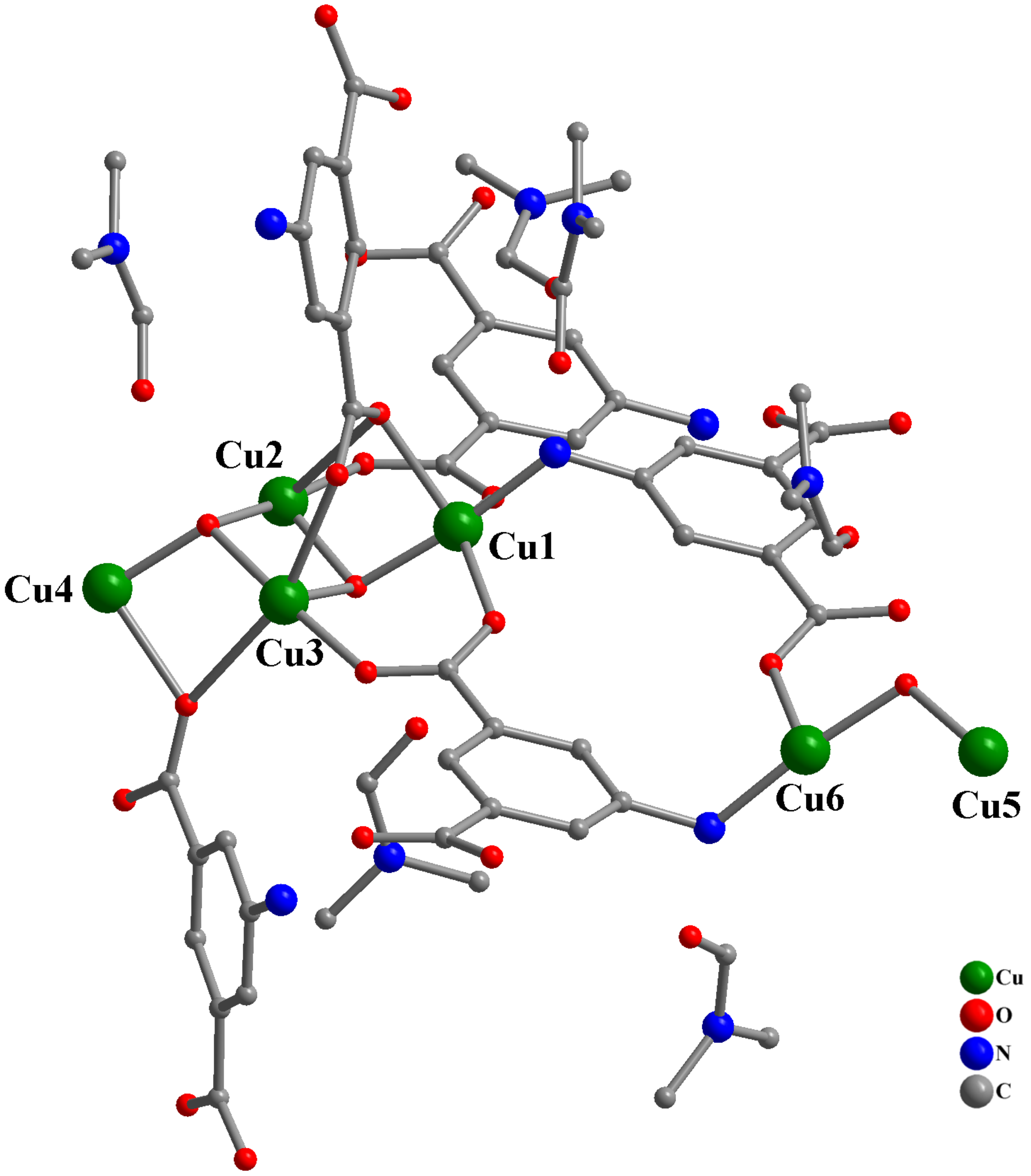

b)

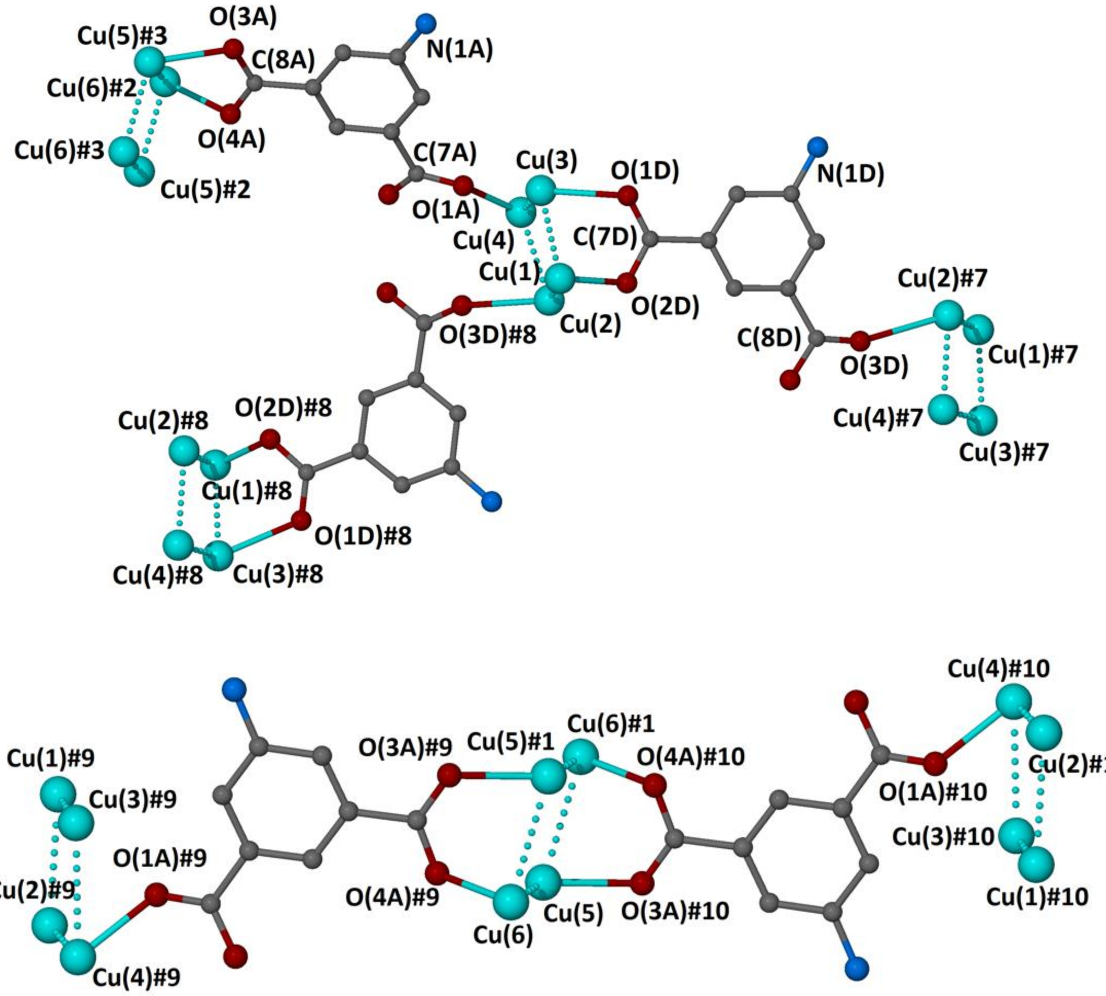

Figure S1: a) The asymmetric unit of **UCY-16**·6nDMF·nH_2O (The lattice water molecule and hydrogen atoms are omitted), b) The connectivity of the two tetranuclear butterfly-like SBUs in **UCY-16**·6nDMF·nH_2O, above and below *ac* plane. Symmetry operations to generate equivalent atoms: #1, -x+2, -y+1, -z; #2, x-1/2, -y+3/2, -z; #3, -x+3/2, y+1/2, z; #7, -x+1, y-1/2, -z+1/2; #8, -x+1, y+1/2, -z+1/2; #9, x+1/2, -y+3/2, -z; #10, -x+3/2, y-1/2, z.

Topologically, ligands **A** and **D** can be obviously considered as linkers which connect adjacent layers. The butterfly-like SBUs, which were six connected nodes in the kgd net have now different connectivity; butterfly Cu(1) – Cu(4) is a nine connected node while butterfly Cu(5) – Cu(6) is an eight connected node. Eventually, though the connectivity of the three connected nodes (ligands **B**, **C** and **E**) remains the same, their topological description changes (related to the **kgd** net) and the net is transformed to a tetranodal one with stoichiometry $(3c)_4(3c)_2(8c)(9c)_2$.

The topology of the network is new, and its point symbol is $\{4^3\}_6\{4^{10}.6^{15}.8^3\}\{4^9.6^{21}.8^6\}_2$. The differences in the 3c nodes can be seen in the extended point symbol of the net which is $[4.4.4(2)]_4[4.4.4]_2$[4.4.4.4.4.4.4.4.4.4.6(2).6(2).6(2).6(2).6(2).6(2).6(4).6(4).6(4).6(4).6(4).6(6).6(6).6(6).6(6).8(8).8(16).8(16)][4.4.4..4.4.4.4.4(2).6(2).6(2).6(2).6(2).6(2).6(2).6(2).6(3).6(3).6(4).6(4).6(4).6(4).6(5).6(5).6(5).6(5).6(6).6(6).6(6).6(6).8(8).8(19).8(19).8(28).8(28).8(37)$]_2$.

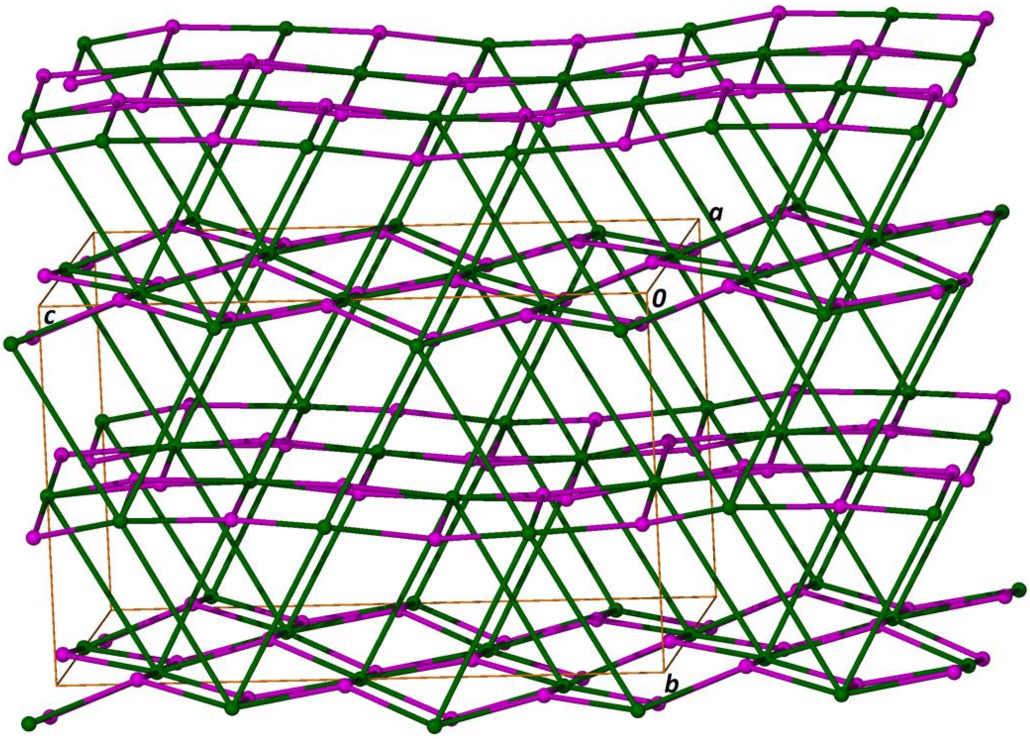

Figure S2: The topological network formed in **UCY-16**·6nDMF·nH_2O with point symbol $\{4^3\}_6\{4^{10}.6^{15}.8^3\}\{4^9.6^{21}.8^6\}_2$. Color code: C, grey; O, red; N, blue; Cu, sky blue; butterfly centroid, green; AIP aromatic ring centroid, pink

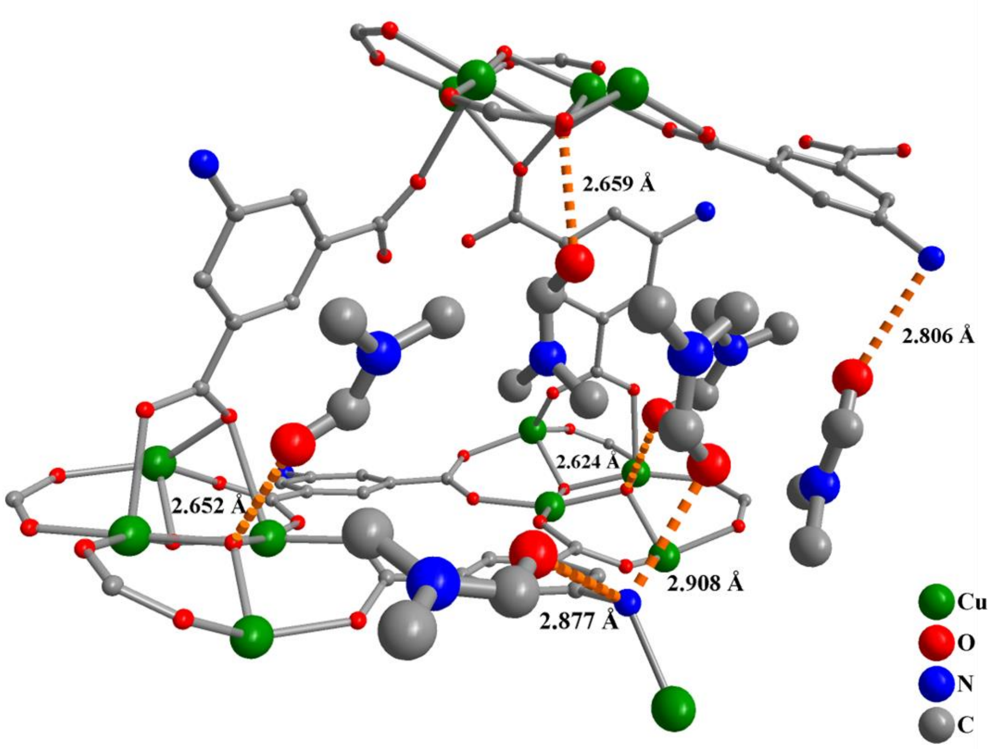

Figure S3: Part of the framework of **UCY-16**·6nDMF·nH_2O (ball and stick model) emphasizing on the hydrogen bonding interactions (dashed orange lines) between the lattice DMF molecules (in enlarged ball and stick model) and the framework. Hydrogen atoms are omitted for clarity.

Physical Measurements/Characterization of UCY-16·6nDMF·nH_2O

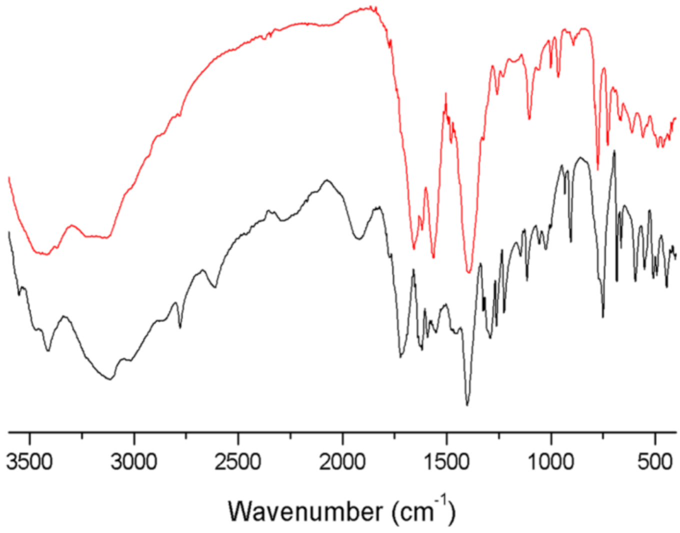

Figure S4: IR spectra of H_2AIP (black) and the as synthesized compound **UCY-16**·6nDMF·nH_2O (red).

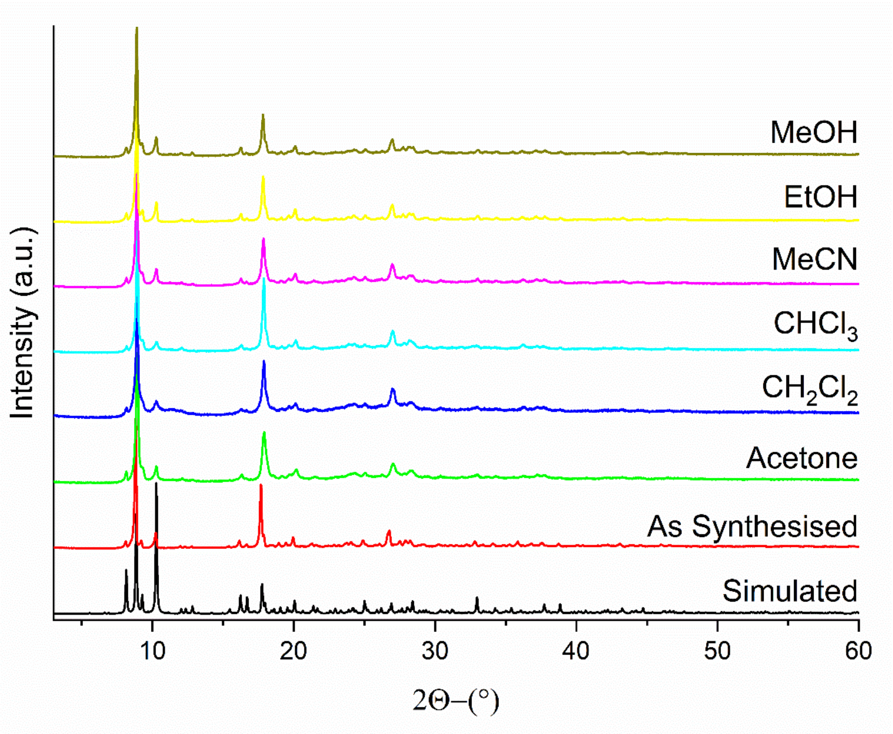

Figure S5: Powder X-ray diffraction patterns of the as synthesized compound **UCY-16**·6nDMF·nH_2O, along with the simulated pattern from single crystal data and the as synthesized **UCY-16**·6nDMF·nH_2O treated (as described in the experimental part) in the indicated organic solvents for 3 days.

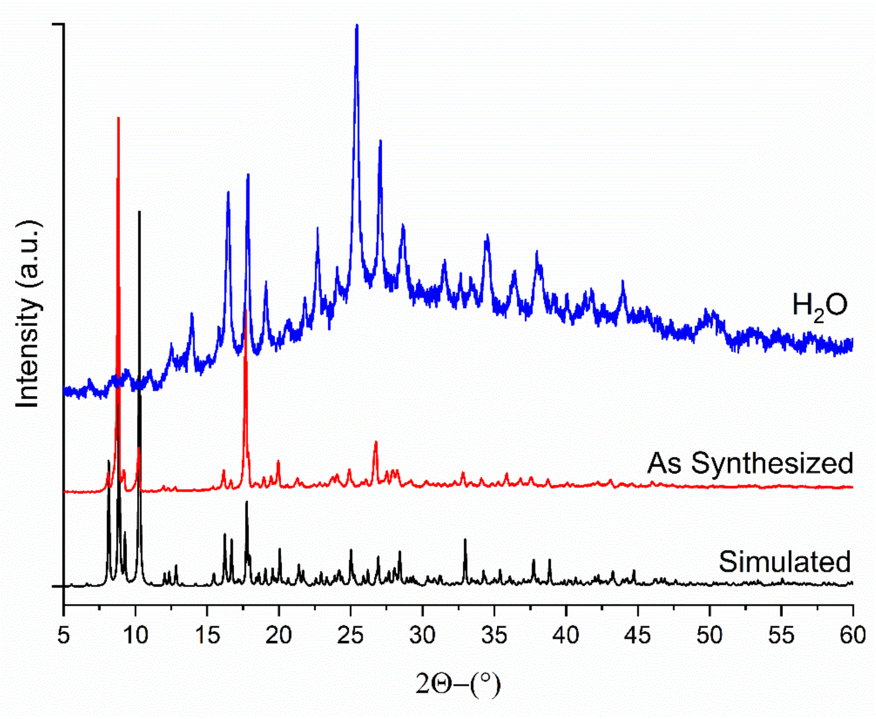

Figure S6: Powder X-ray diffraction pattern of the as synthesized **UCY-16**·6nDMF·nH_2O treated (as described in the experimental part) in water for 1 day.

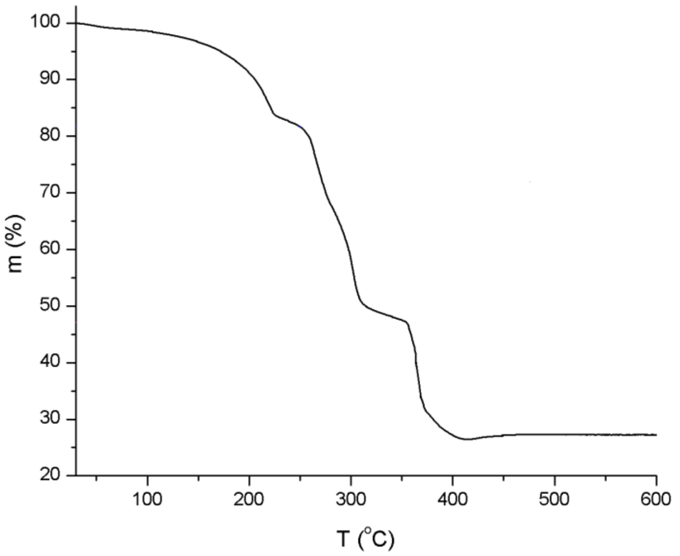

Figure S7: TGA graph of the as synthesized compound **UCY-16**·6nDMF·nH_2O. The decomposition of **UCY-16**·6nDMF·nH_2O is a multistep process which begins with the slow removal of the lattice solvent molecules from 20°C up to ~270 °C (calculated loss ≈ 25.6%; found ≈ 25%). The lattice solvent molecule loss is followed by the decomposition of the framework that involves the removal of the AIP^{2-}/$HAIP^{-}$ ligands which is completed at ~450 °C (calculated loss ≈ 47.7%; found ≈ 48%). The residue at 600 °C corresponds to CuO (calculated residue ≈ 26.7%; found ≈ 27%).

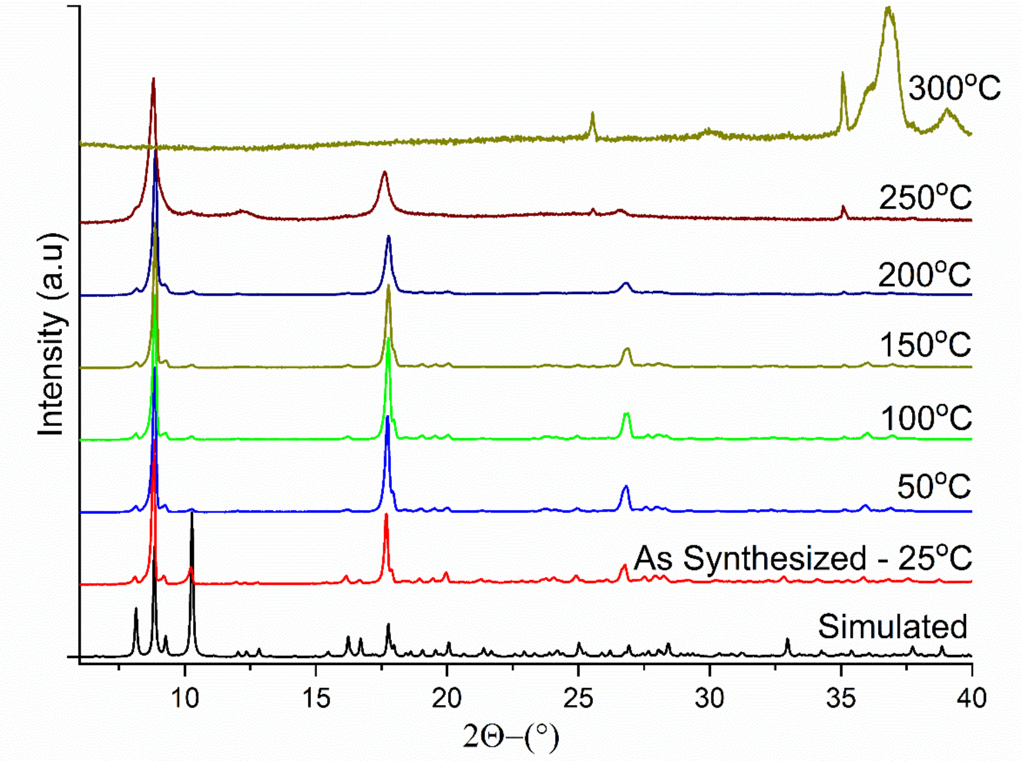

Figure S8: Variable temperature powder X-ray diffraction patterns of the as synthesized **UCY-16**·6nDMF·nH_2O recorded under Ar flow.

Structural figures of UCY-16/S (S = Bz, Tol, PhCl and MeCN)

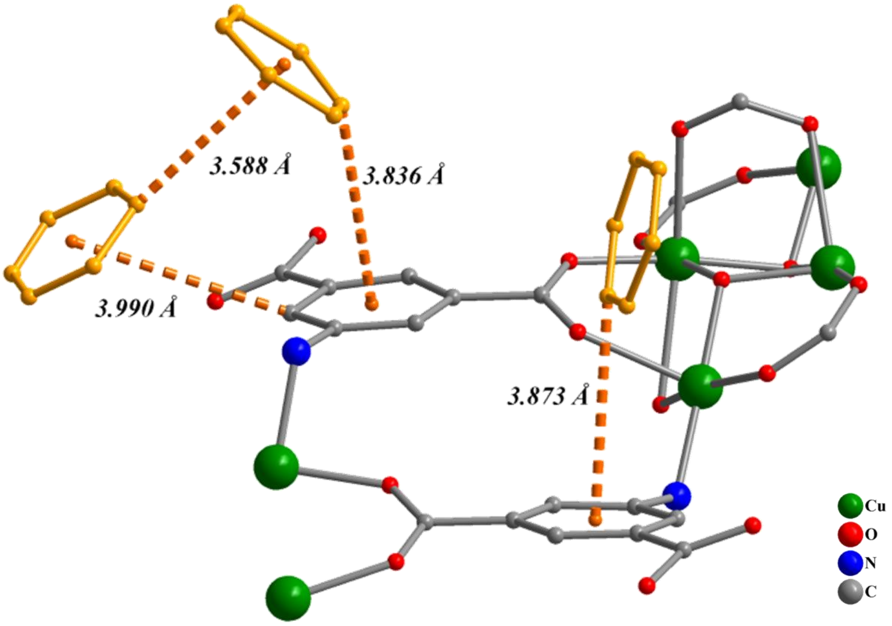

Figure S9: Part of the framework of **UCY-16**/Bz (ball and stick model) emphasizing on selected π···π edge to face interactions (dashed orange lines) between the lattice benzene molecules (in orange) and the framework. Hydrogen atoms are omitted for clarity.

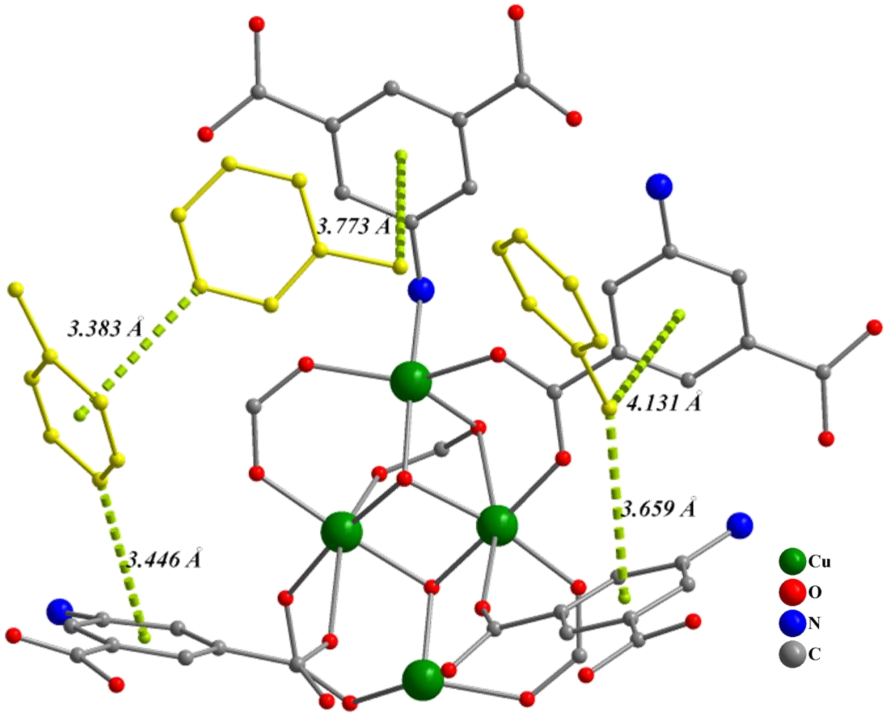

Figure S10: Part of the framework of **UCY-16**/Tol (ball and stick model) emphasizing on selected π···π edge to face and CH_3···π interactions (dashed green lines) between the lattice toluene molecules (in yellow) and the framework. Hydrogen atoms are omitted for clarity.

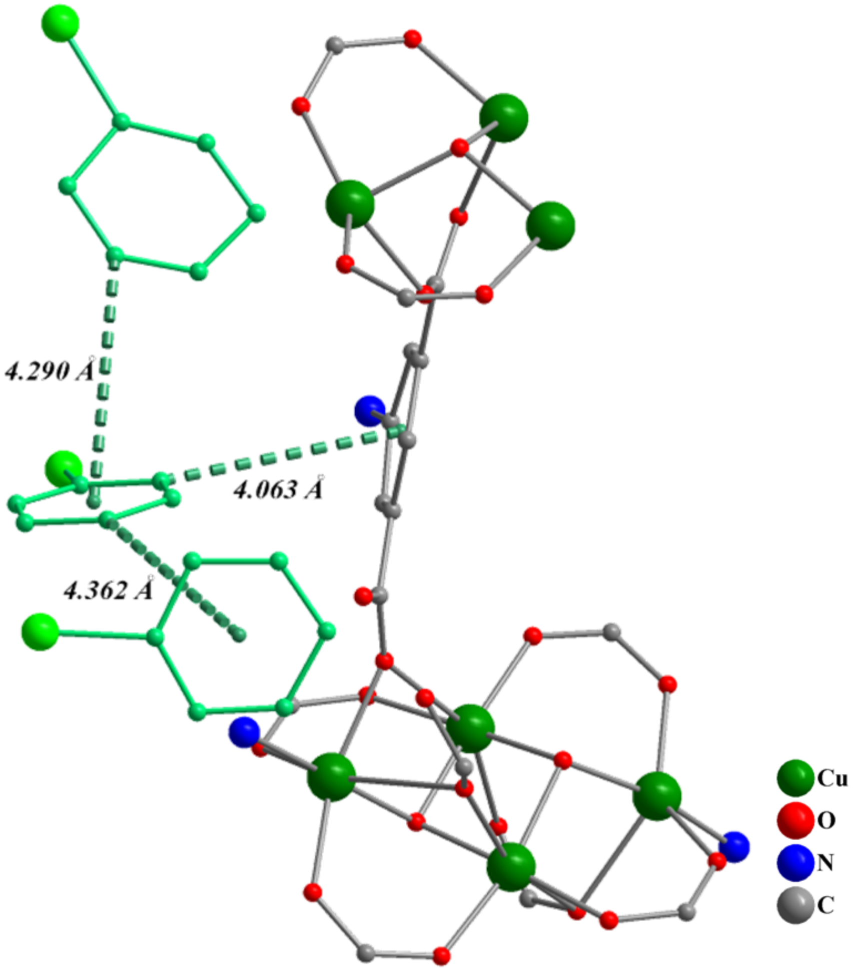

Figure S11: Part of the framework of **UCY-16**/PhCl (ball and stick model) emphasizing on selected π···π stacking edge to face interactions (dashed green lines) between the lattice chlorobenzene molecules (in green) and the framework. Hydrogen atoms are omitted for clarity.

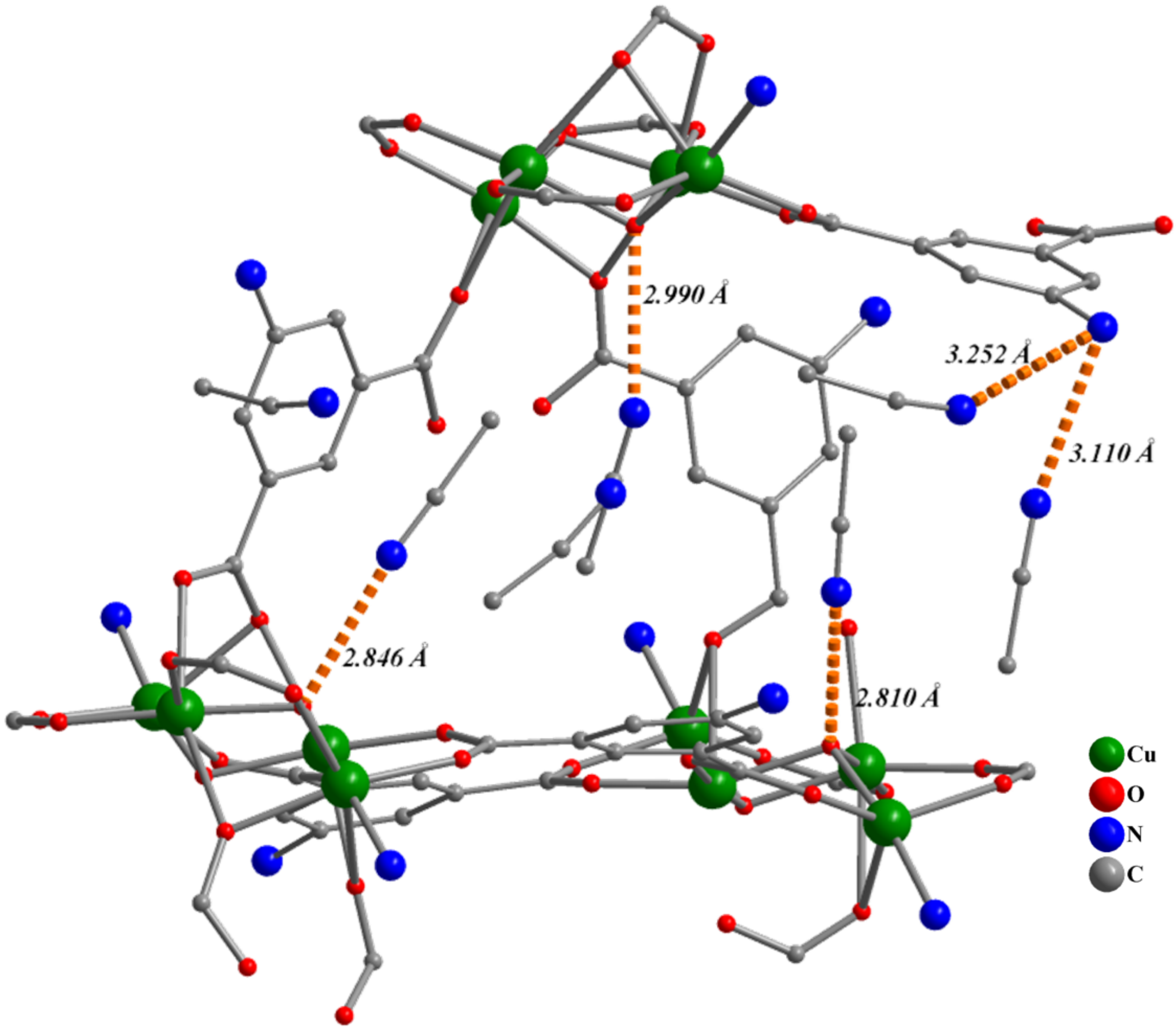

Figure S12: Part of the framework of **UCY-16**/MeCN (ball and stick model) emphasizing on selected hydrogen bonding interactions (dashed orange lines) between the lattice MeCN molecules and the framework. Hydrogen atoms are omitted for clarity.

Physical Measurements/Characterization of compounds UCY-16/S (S = Bz, Tol, PhCl and MeCN)

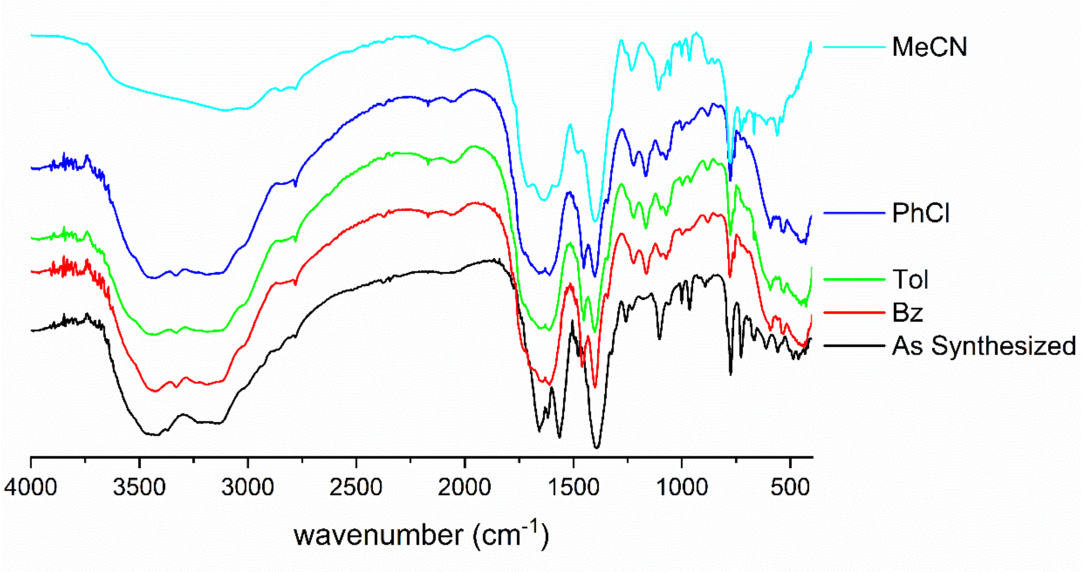

Figure S13: IR spectra of the as synthesized compound **UCY-16**·6nDMF·nH_2O and the exchanged analogues **UCY-16**/S (S = Bz, Tol, PhCl, MeCN).

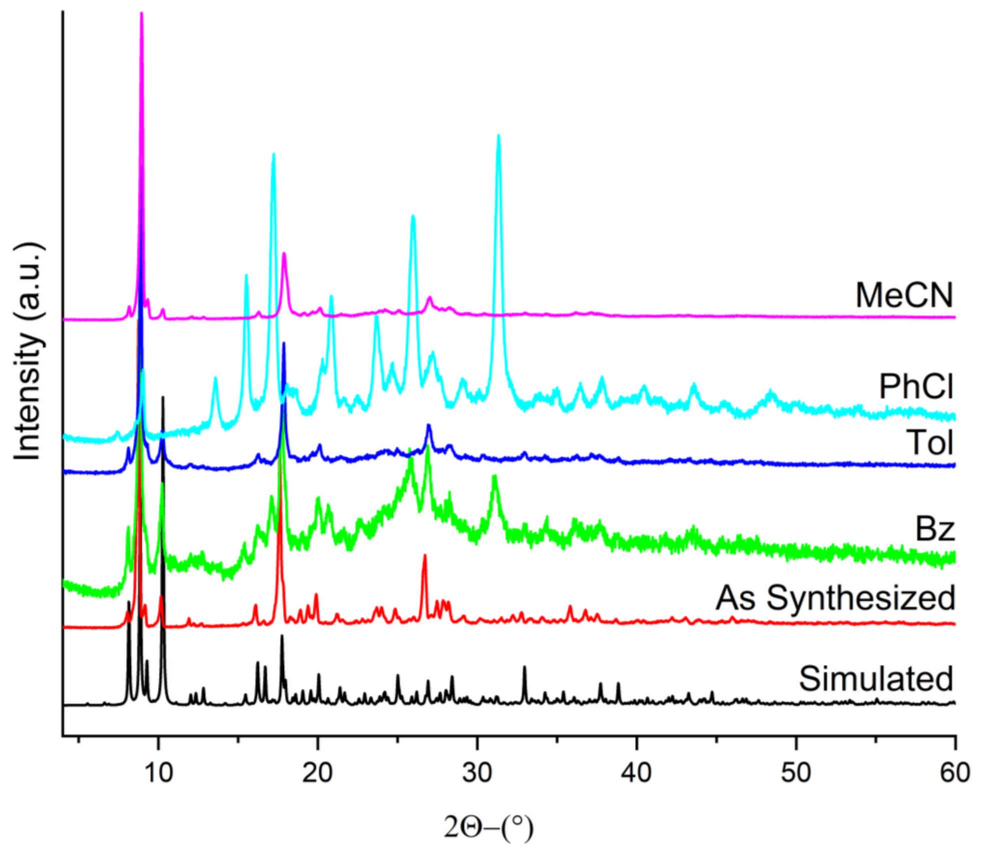

Figure S14: pXRD patterns of the as synthesized compound **UCY-16**·6nDMF·nH_2O and the exchanged analogues **UCY-16**/S (S = Bz, Tol, PhCl, MeCN).

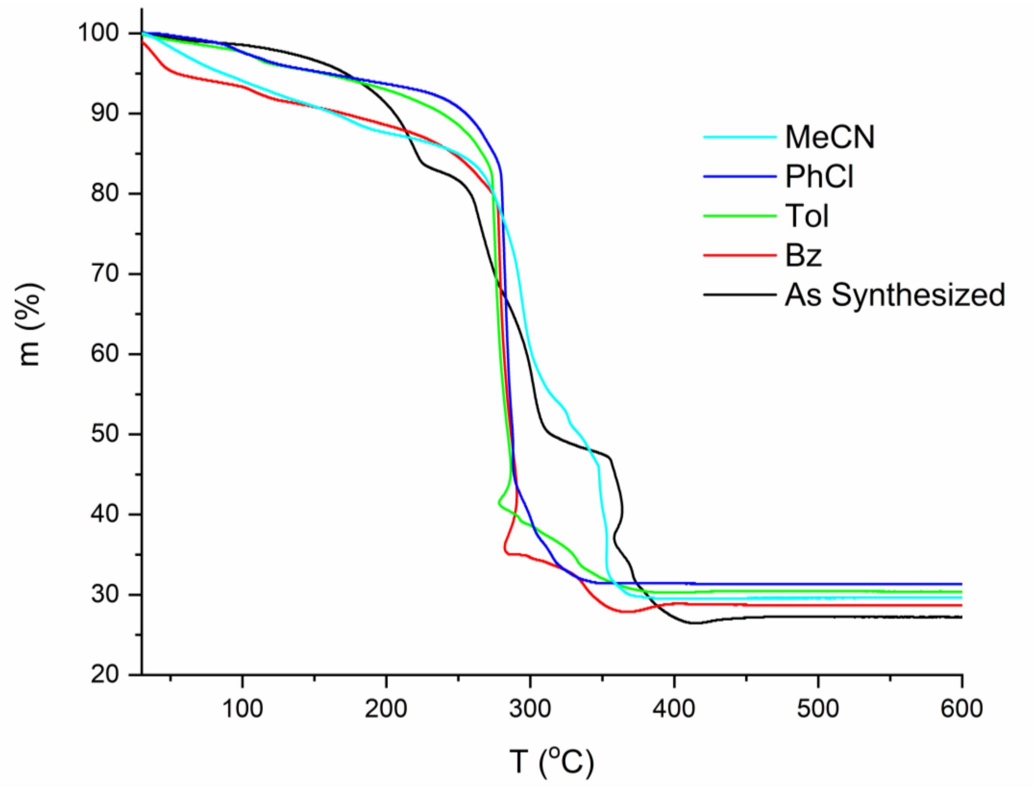

Figure S15: TGA graphs of the as synthesized compound **UCY-16**·6nDMF·nH_2O and the exchanged analogues **UCY-16**/S (S = Bz, Tol, PhCl, MeCN). TG analysis revealed that the decomposition of **UCY-16**/S (S = Bz, Tol, PhCl, MeCN) is completed in two steps. The first one is attributed to the release of lattice solvent molecules and residual water or DMF molecules and is completed at 255-275°C whereas the second one is completed at 435-465°C and is attributed to the combustion of AIP^{2-}/$HAIP^{-}$ ligands. Finally, the residue at 600°C corresponds to CuO. (Table S3)

Table S3: Calculated values for solvent removal and ligand combustion along with the experimental values obtained from TG analysis of the compounds **UCY-16**·6nDMF·nH_2O and **UCY-16**/S (S = Bz, Tol, PhCl, MeCN).

	Solvent Content			Ligand			Residue (at 600°C)	
Compound	θ(°C)	Calc.	Exp.	θ(°C)	Calc.	Exp.	Calc.	Exp.
**UCY-16**·3Bz·DMF·2H_2O	275	20	21	435	51	49	29	30
**UCY-16**·3nTol·nDMF·2H_2O	270	22	20	440	50	49	29	31
**UCY-16**·3PhCl·H_2O	280	21	19	435	51	50	28	31
**UCY-16**·6MeCN·2H_2O	255	17	16	465	53	54	30	30

Structural figures of (UCY-16/n-C_xH_{2x+1}OH)·S′ (x = 1-7; S′ = lattice solvents)

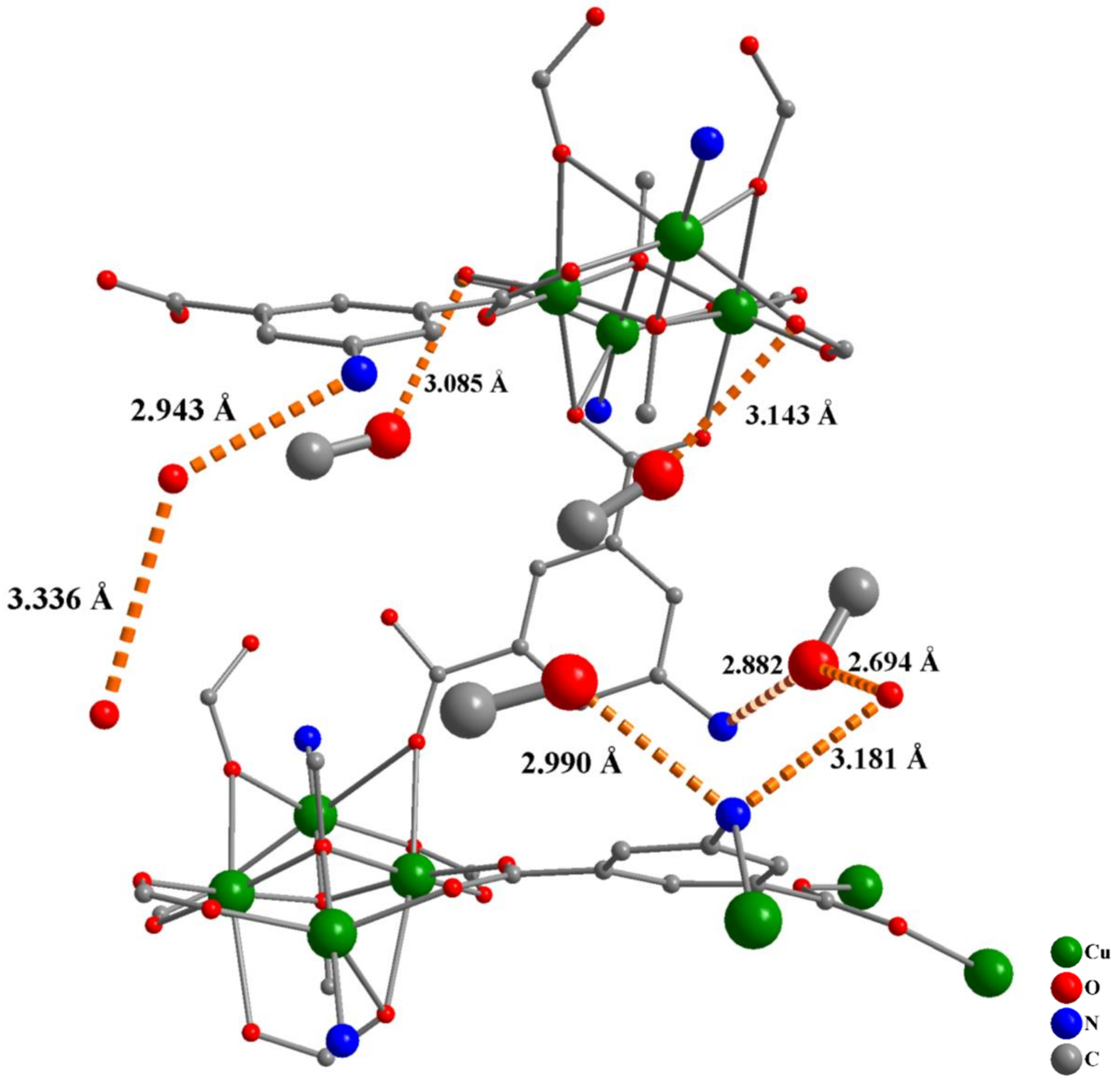

Figure S16: Part of the framework of **UCY-16**/CH_3OH (ball and stick model) emphasizing on the hydrogen bonding interactions (dashed orange lines) between the lattice CH_3OH/H_2O molecules and the framework. Hydrogen atoms are omitted for clarity.

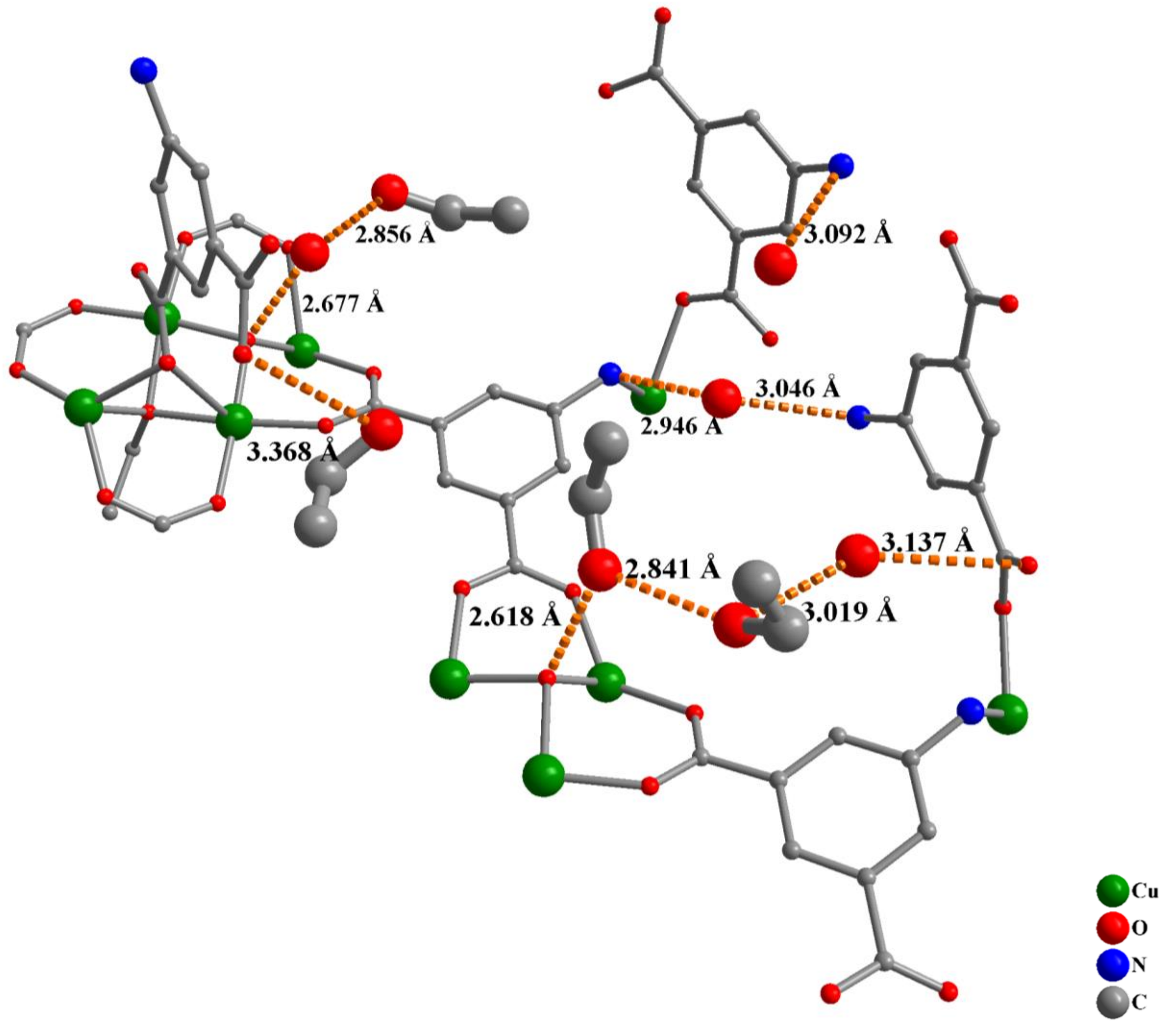

Figure S17: Part of the framework of **UCY-16**/C_2H_5OH (ball and stick model) emphasizing on the hydrogen bonding interactions (dashed orange lines) between the lattice C_2H_5OH/H_2O molecules and the framework. Hydrogen atoms are omitted for clarity.

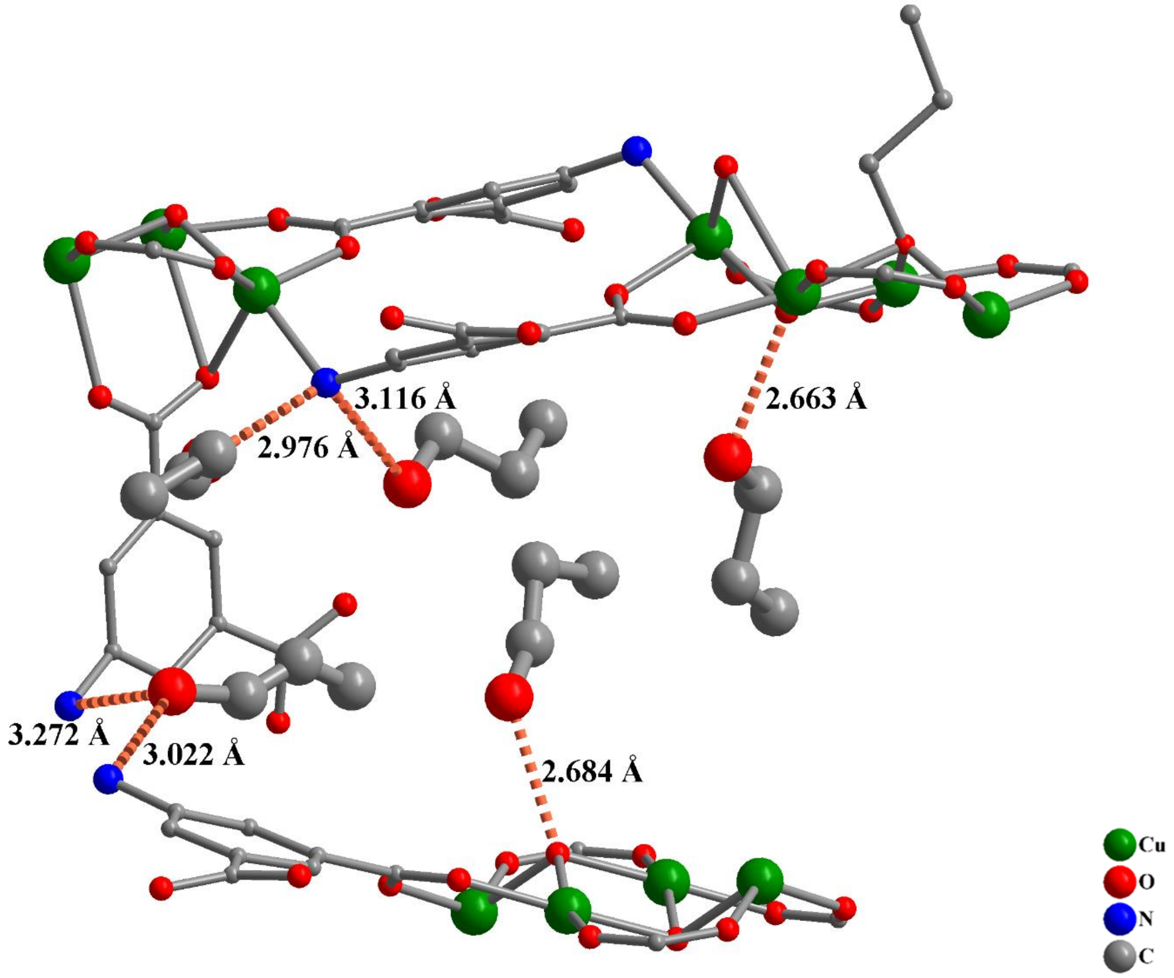

Figure S18: Part of the framework of **UCY-16**/n-C_3H_7OH (ball and stick model) emphasizing on the hydrogen bonding interactions (dashed orange lines) between the lattice n-C_3H_7OH molecules and the framework. Hydrogen atoms are omitted for clarity.

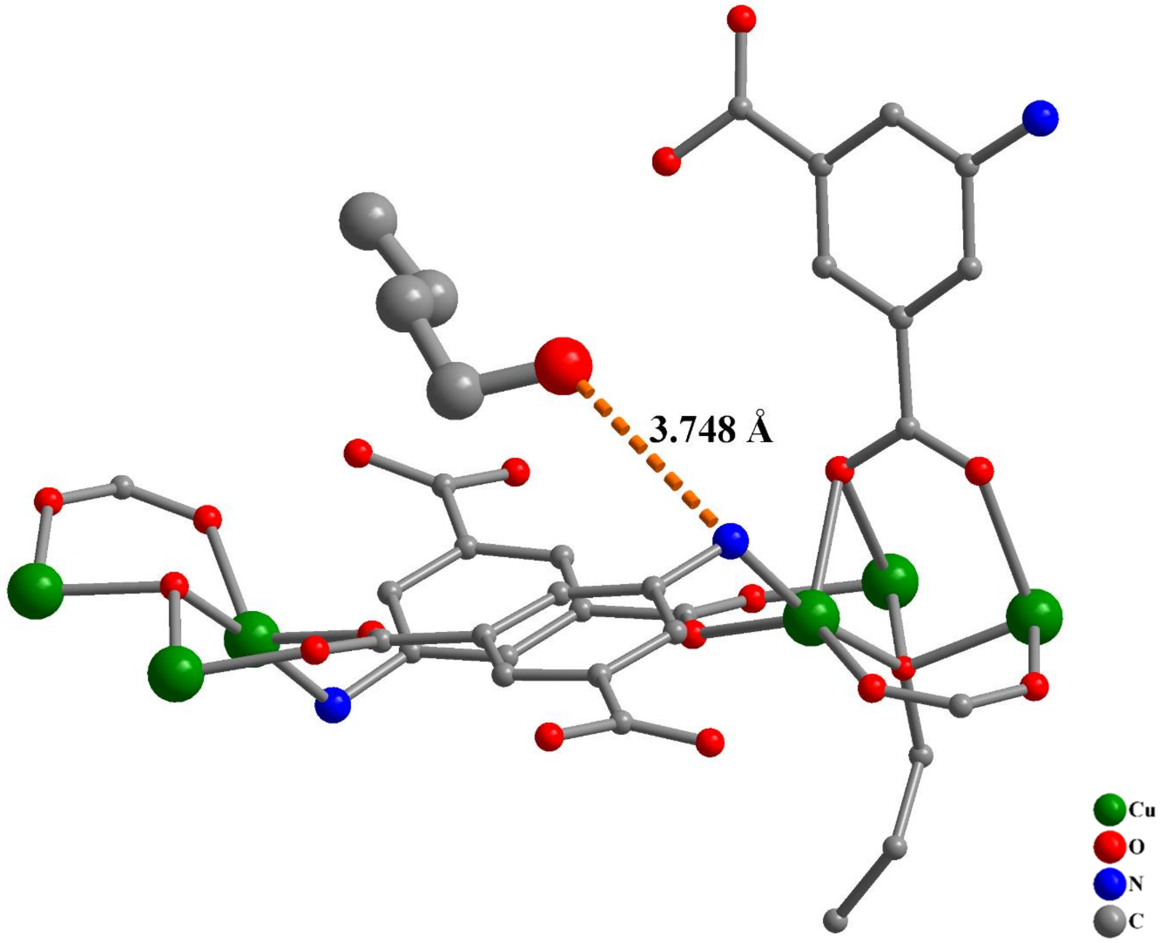

Figure S19: Part of the framework of **UCY-16**/n-C_4H_9OH (ball and stick model) emphasizing on the hydrogen bonding interactions (dashed orange lines) between the lattice n-C_4H_9OH molecule and the framework. Hydrogen atoms are omitted for clarity.

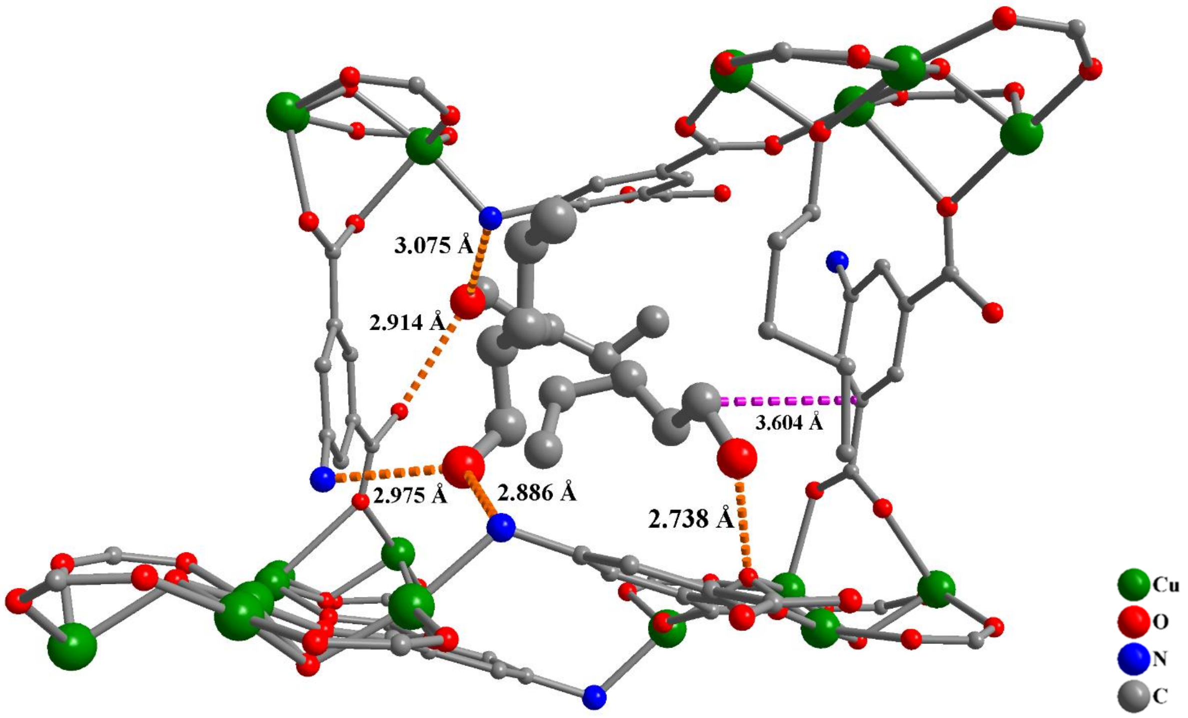

Figure S20: Part of the framework of **UCY-16**/n-$C_5H_{11}OH$ (ball and stick model) emphasizing on the hydrogen bonding (dashed orange lines) and Van der Waals (dashed magenta lines) interactions between the lattice n-$C_5H_{11}OH$ molecules and the framework. Hydrogen atoms are omitted for clarity.

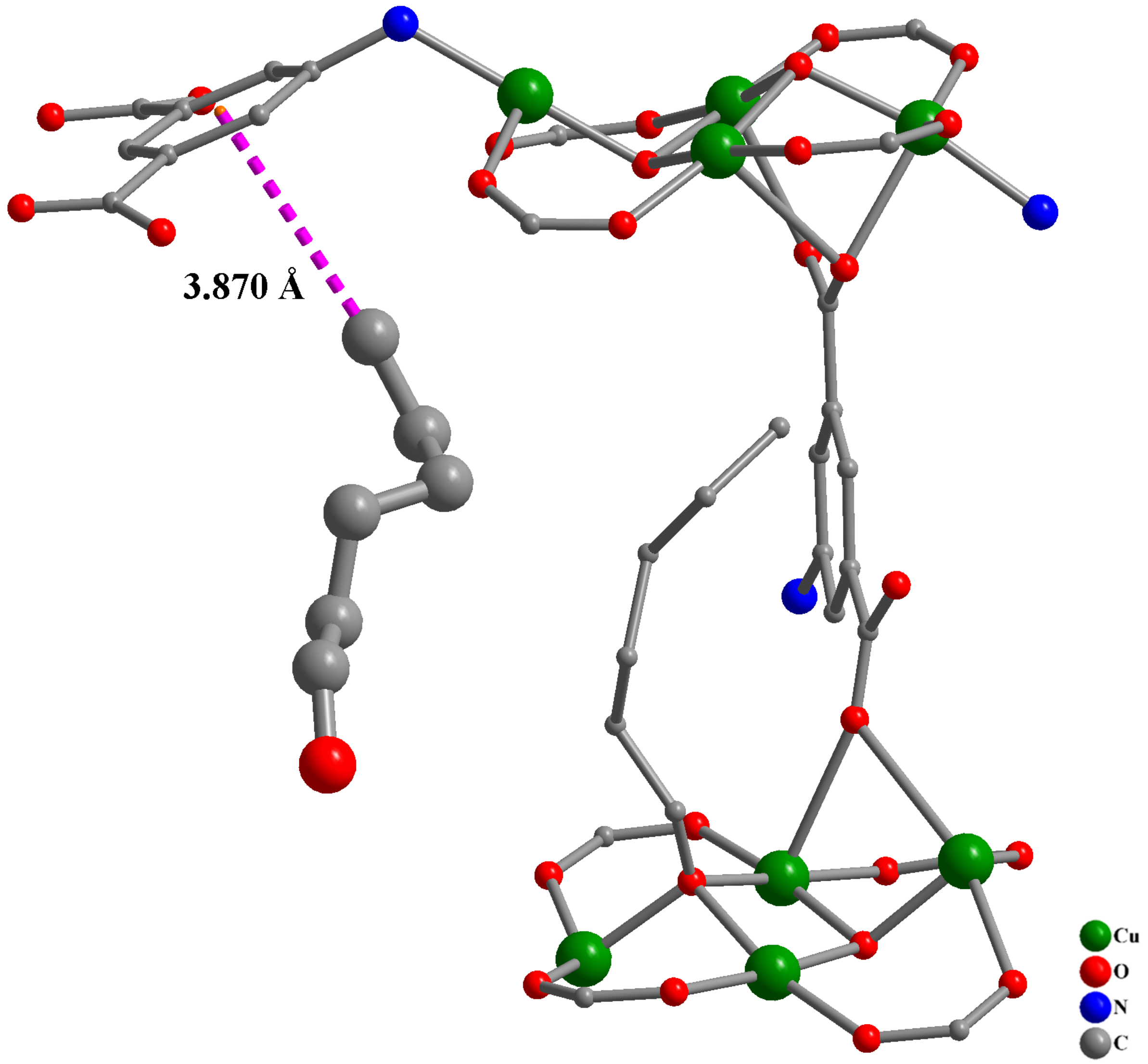

Figure S21: Part of the framework of **UCY-16**/n-$C_6H_{13}OH$ (ball and stick model) emphasizing on the Van der Waals interactions (dashed magenta lines) between the lattice n-$C_6H_{13}OH$ molecule and the framework. Hydrogen atoms are omitted for clarity.

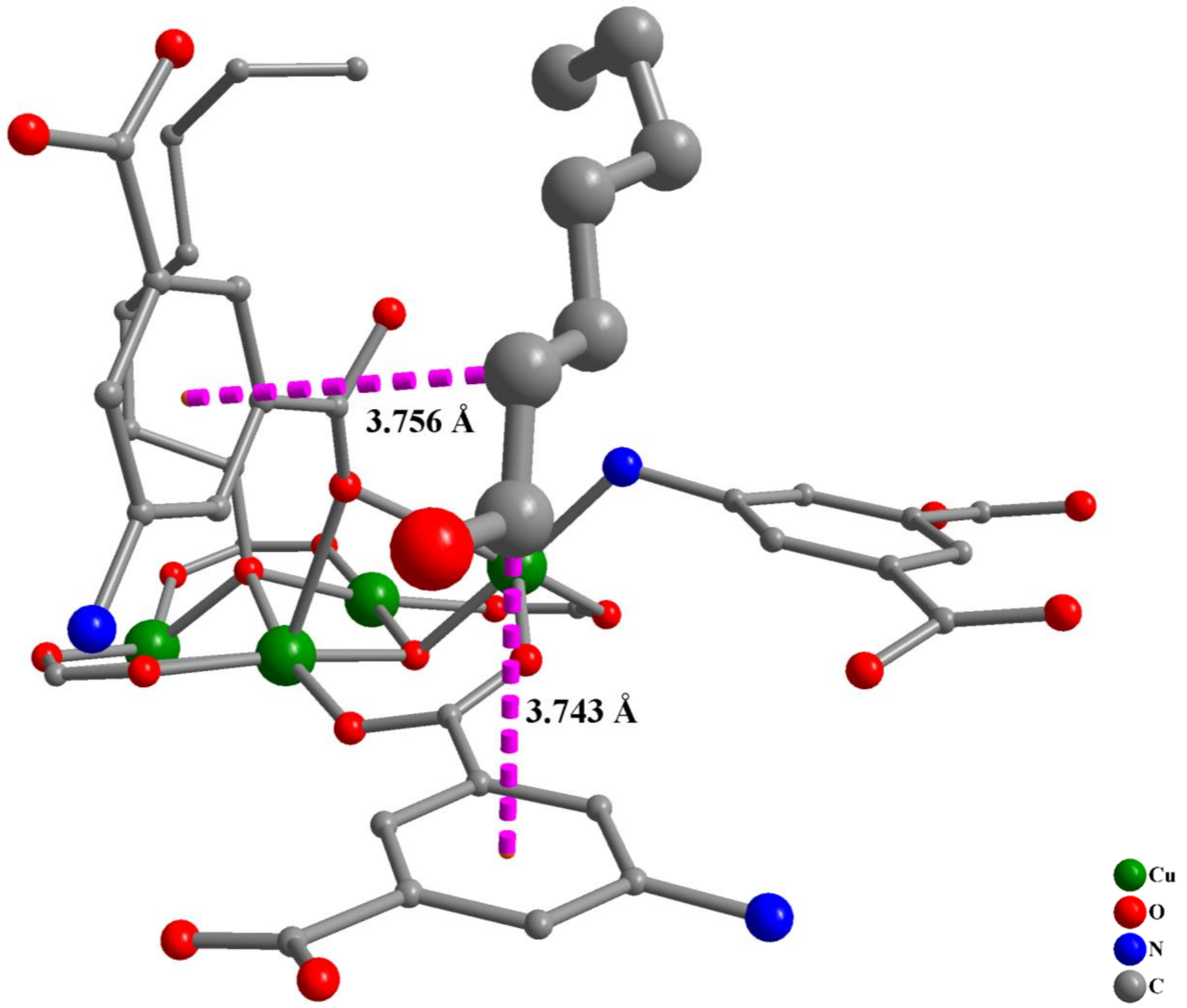

Figure S22: Part of the framework of **UCY-16**/n-$C_7H_{15}OH$ (ball and stick model) emphasizing on the Van der Waals interactions (dashed magenta lines) between the lattice n-$C_7H_{15}OH$ molecule and the framework. Hydrogen atoms are omitted for clarity.

Physical Measurements/Characterization of compounds of (UCY-16/n-$C_xH_{2x+1}OH$)·S′ (x = 1-10, 12, 14 and 16; S′ = lattice solvents)

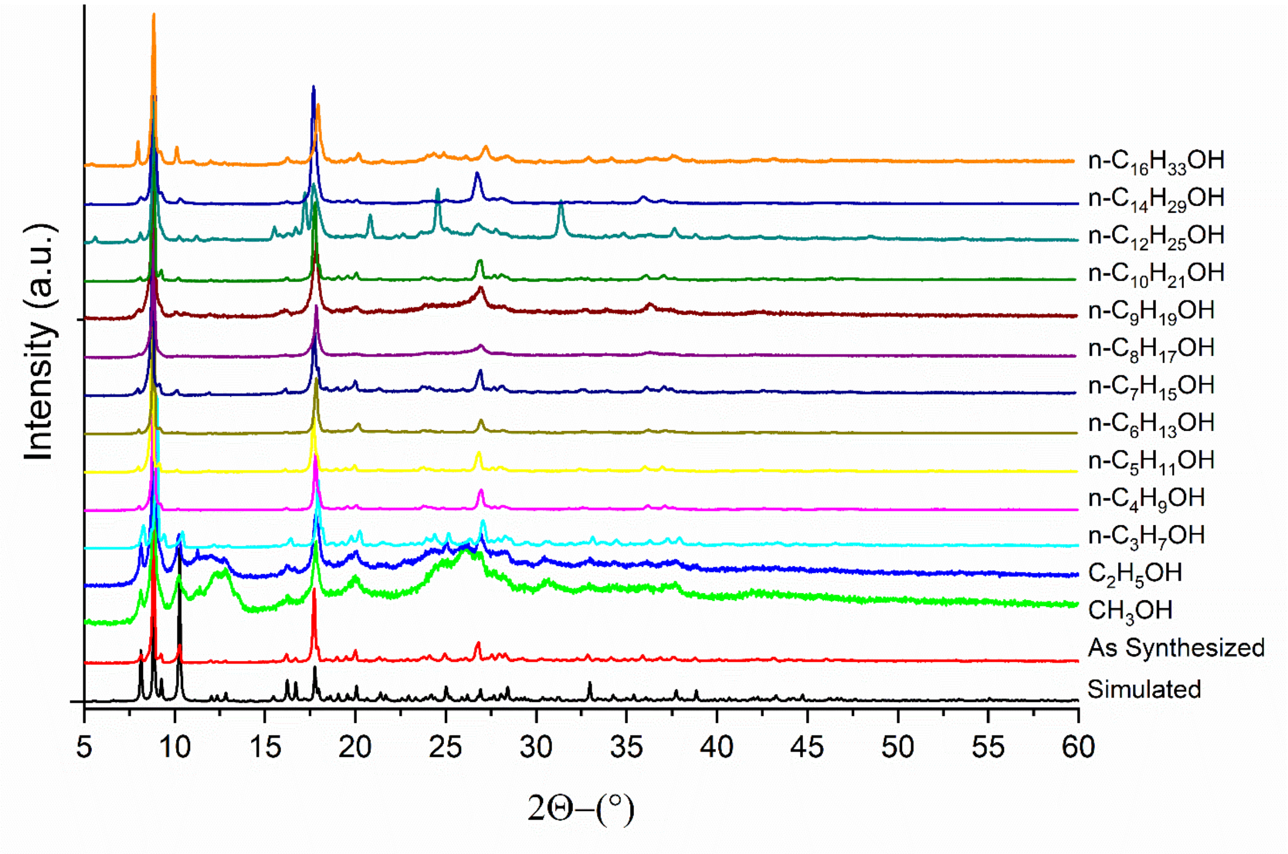

Figure S23: pXRD patterns of the as synthesized compound **UCY-16**·6nDMF·nH_2O and the exchanged analogues **UCY-16**/n-$C_xH_{2x+1}OH$·S′ (x = 1-10, 12, 14 and 16).

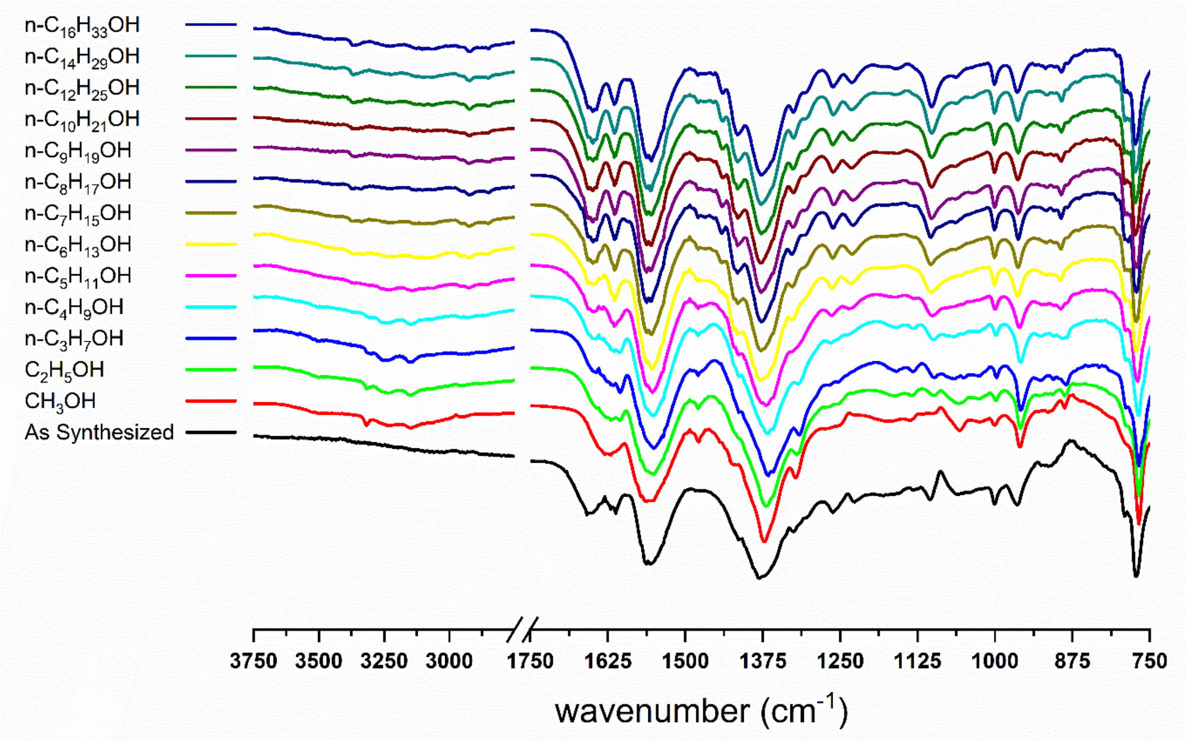

Figure S24: IR spectra of the as synthesized compound **UCY-16**·6nDMF·nH_2O and the exchanged analogues **UCY-16**/n-$C_xH_{2x+1}OH$·S′ (x = 1-10, 12, 14 and 16).

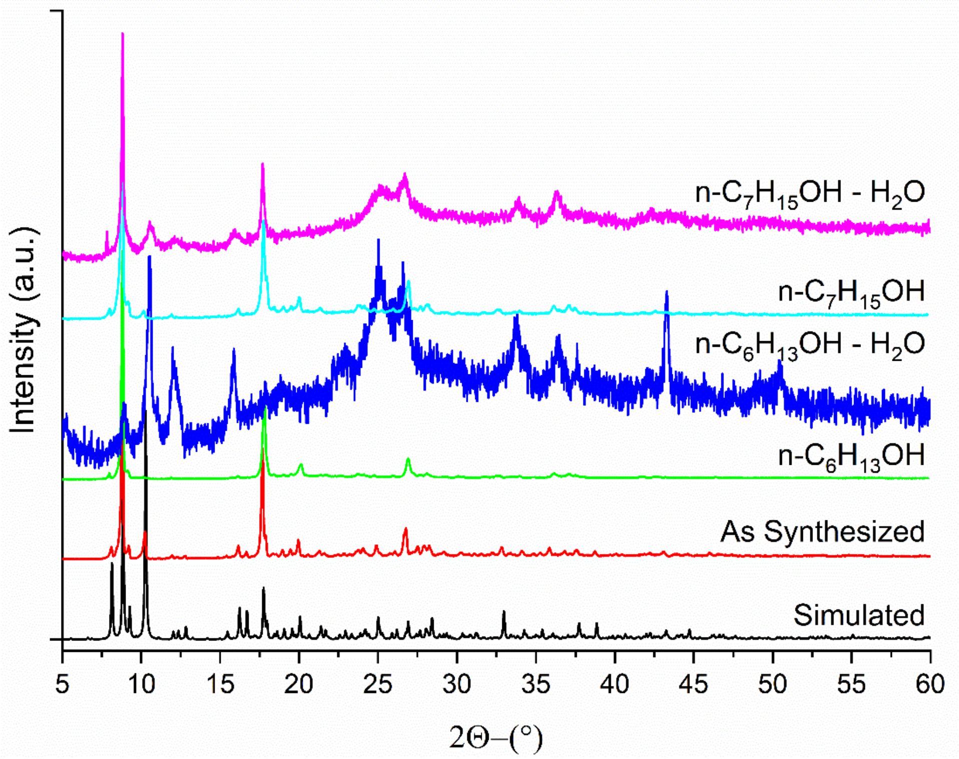

Figure S25: pXRD patterns of the exchanged analogues **UCY-16**/n-$C_xH_{2x+1}OH$·S′ (x = 6 and 7) treated (as described in the experimental part) in water for 1 day.

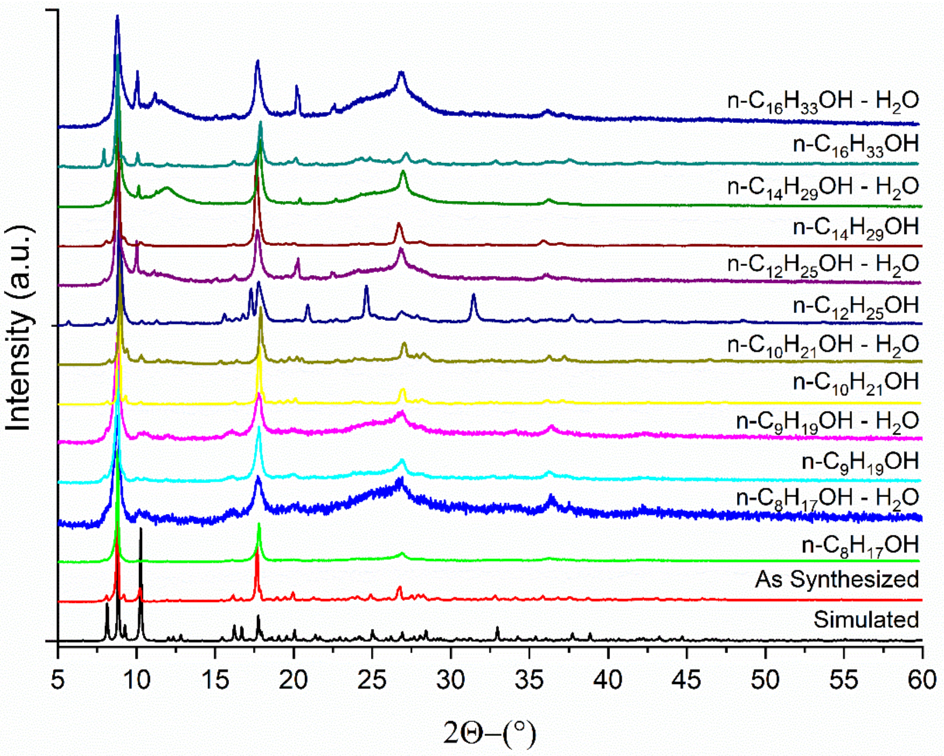

Figure S26: pXRD patterns of the exchanged analogues **UCY-16**/n-$C_xH_{2x+1}OH \cdot S'$ (x = 8-10, 12, 14 and 16) treated (as described in the experimental part) in water for 1 day.

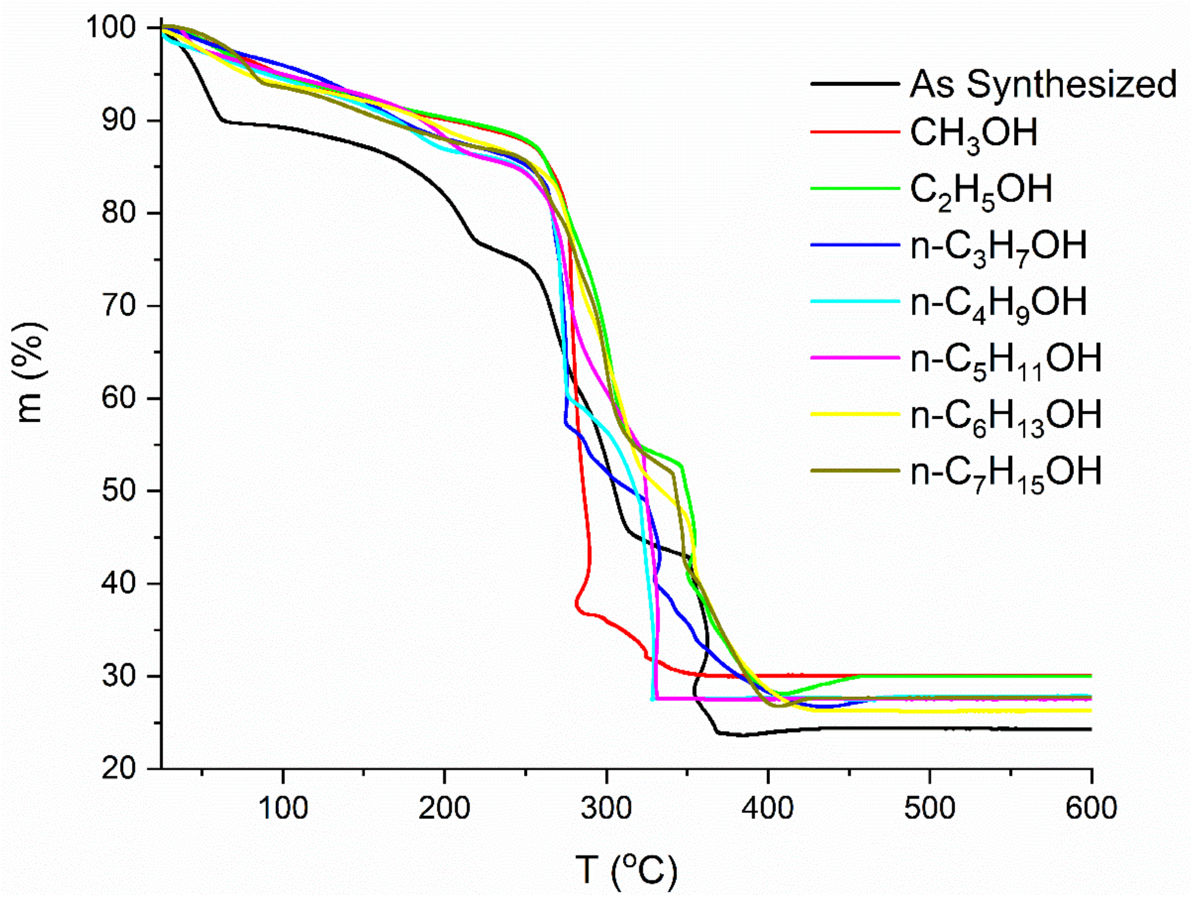

Figure S27: TGA graphs of the as synthesized compound **UCY-16**·6nDMF·nH_2O and **UCY-16**/n-$C_xH_{2x+1}OH$·S′ (x = 1-7). TG analysis revealed that the decomposition of the exchanged analogues **UCY-16**/n-$C_xH_{2x+1}OH$·S′ (x = 1-7) is completed in two steps. The first step is attributed to the release of lattice alcohol molecules and residual water or DMF lattice solvents and is completed at 260-270°C whereas the second step is completed at 500°C and is attributed to the combustion of AIP^{2-}/$HAIP^{-}$/n-$C_xH_{2x+1}O^{-}$ ligands. Finally, the residue at 600°C corresponds to CuO. (Table S4)

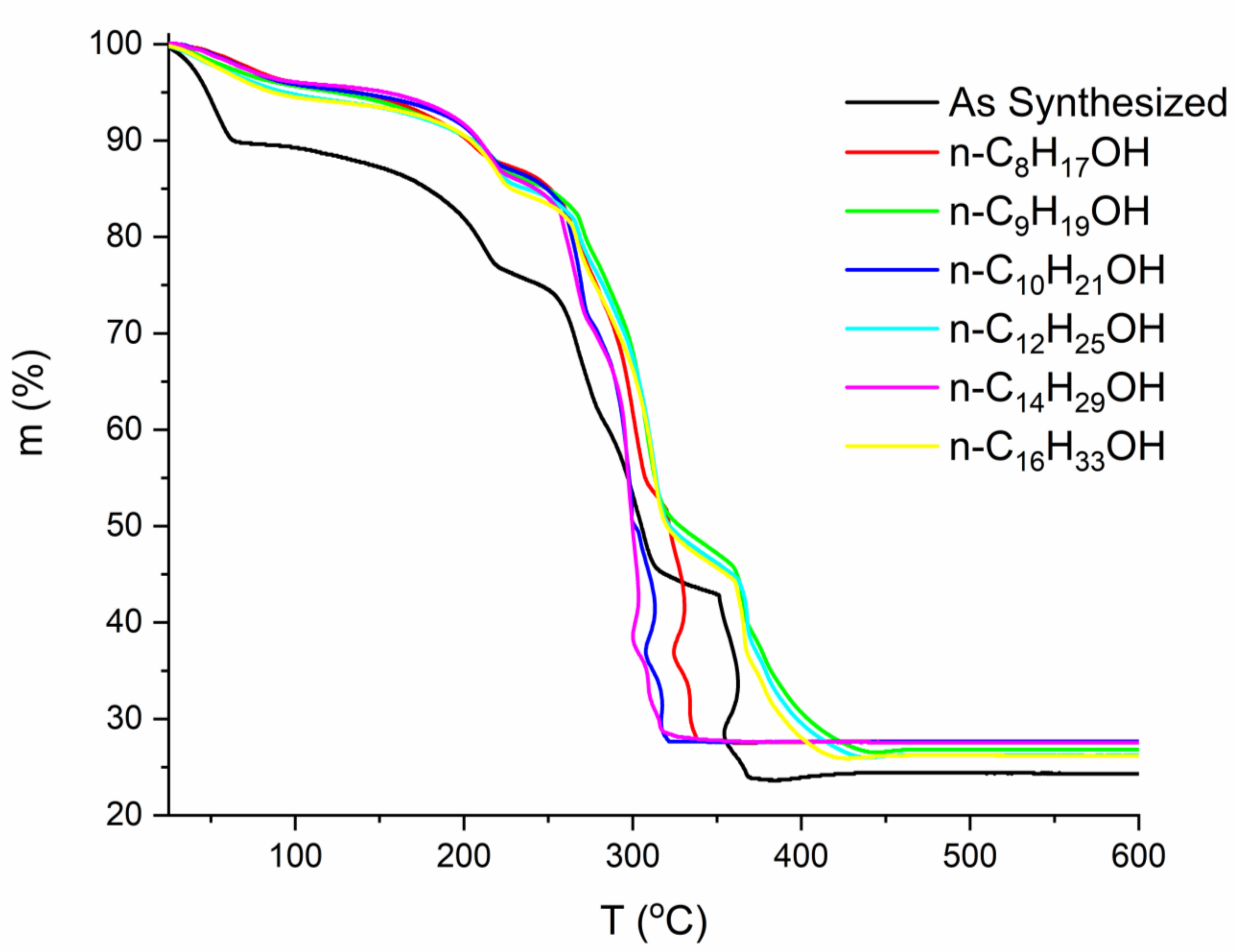

Figure S28: TGA graphs of the as synthesized compound **UCY-16**·6nDMF·nH_2O and **UCY-16**/n-C_xH_{2x+1}OH·S′ (x = 8-10, 12, 14 and 16). TG analysis revealed that the decomposition of the exchanged analogues **UCY-16**/n-C_xH_{2x+1}OH·S′ (x = 8-10, 12, 14 and 16) is completed in two steps. The first step is attributed to the release of lattice alcohol molecules and residual water or DMF lattice solvents and is completed at 250-270°C whereas the second step is completed at 500°C and is attributed to the combustion of AIP^{2-}/$HAIP^{-}$/n-$C_xH_{2x+1}O^{-}$ ligands. Finally, the residue at 600°C corresponds to CuO. (Table S4)

Table S4: Calculated values for solvent removal and ligand combustion along with the experimental values obtained from TG analysis of the compounds **UCY-16**·6nDMF·nH_2O and **UCY-16**/n-$C_xH_{2x+1}OH$·S′ (x = 1-10, 12, 14 and 16)

	Solvent Content			Ligand			Residue (at 600°C)	
Compound	θ(°X)	Theoretical	Experimental	θ(°X)	Theoretical	Experimental	Theoretical	Experimental
(**UCY-16**/CH_3OH)·7CH_3OH·3H_2O	260	17	18	500	54	52	29	30
(**UCY-16**/C_2H_5OH)·7C_2H_5OH·H_2O	260	21	19	500	51	51	28	30
(**UCY-16**/n-C_3H_7OH)·5n-C_3H_7OH	260	18	17	500	53	55	29	28
(**UCY-16**/n-C_4H_9OH)·5n-C_4H_9OH·0.5DMF	270	23	22	500	50	50	27	28
(**UCY-16**/n-$C_5H_{11}OH$)·5n-$C_5H_{11}OH$	270	24	22	500	50	50	26	28
(**UCY-16**/n-$C_6H_{13}OH$)·3n-$C_6H_{13}OH$	270	18	17	500	54	53	28	30
(**UCY-16**/n-$C_7H_{15}OH$)·3n-$C_7H_{15}OH$	270	20	20	500	53	52	27	28
(**UCY-16**/n-$C_8H_{17}OH$)·2n-$C_8H_{17}OH$	250	15	14	500	57	59	28	28
(**UCY-16**/n-$C_9H_{19}OH$)·2n-$C_9H_{19}OH$	250	17	15	500	56	58	27	27
(**UCY-16**/n-$C_{10}H_{21}OH$)·1.5n-$C_{10}H_{21}OH$·1DMF	250	17	15	500	56	57	27	28
(**UCY-16**/n-$C_{12}H_{25}OH$)·1.5n-$C_{12}H_{25}OH$·1DMF	250	19	17	500	55	57	26	26
(**UCY-16**/n-$C_{14}H_{29}OH$)·1n-$C_{14}H_{29}OH$·1DMF	250	16	14	500	58	58	26	28
(**UCY-16**/n-$C_{16}H_{33}OH$)·1n-$C_{16}H_{33}OH$·1DMF	250	17	16	500	58	58	25	26

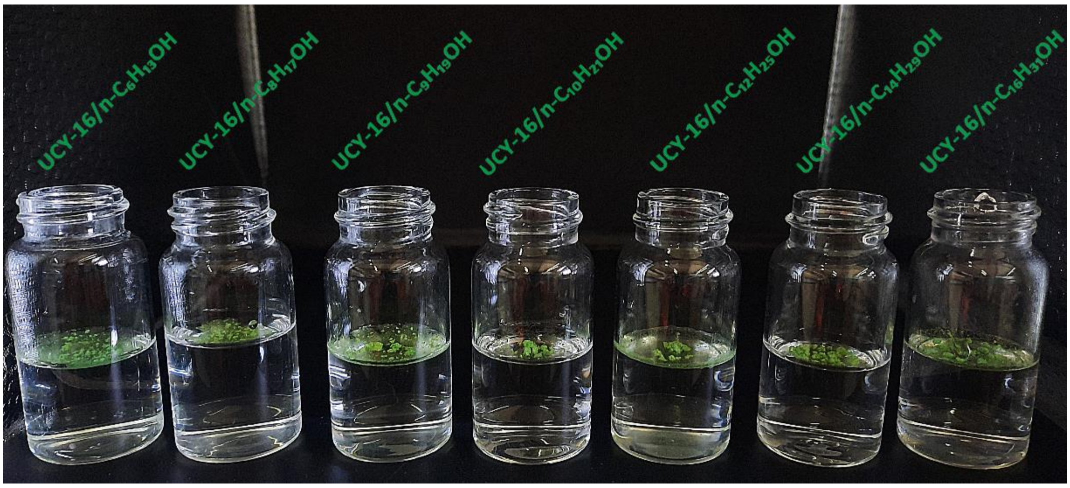

Figure S29: Image of the series of **UCY-16**/n-$C_xH_{2x+1}OH{\cdot}S'$ (x = 6, 8-10, 12, 14, 16) MOF samples floating on the water surface.

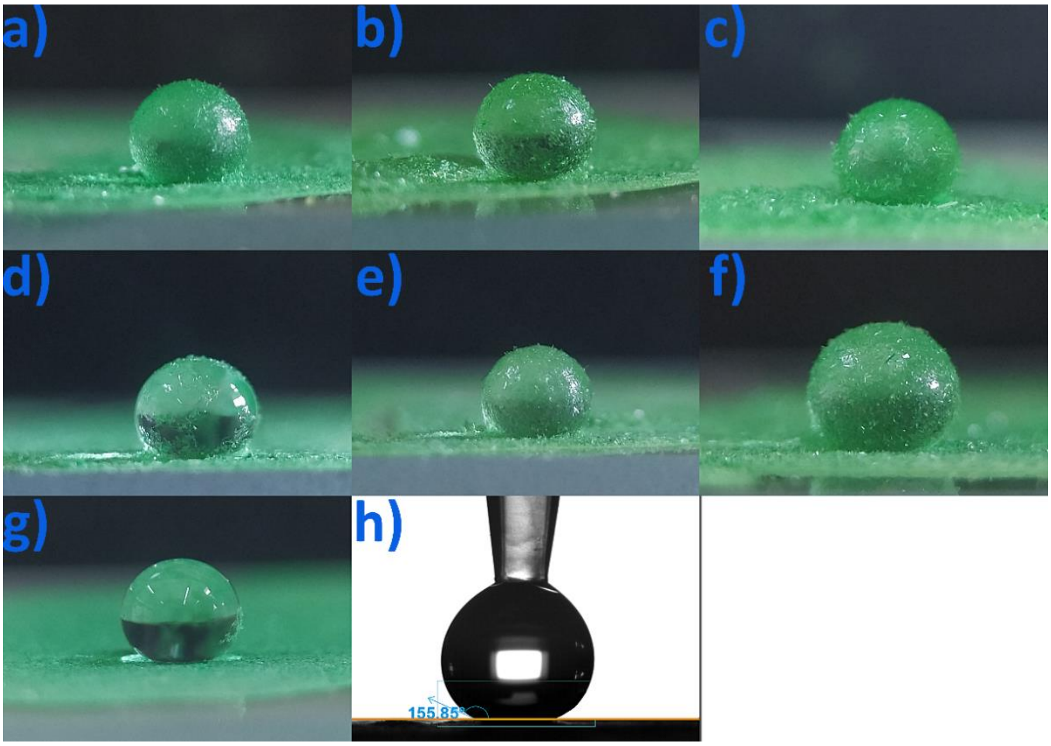

Figure S30: Distilled water droplets (10 μL) on a film of a) **UCY-16**/n-$C_6H_{13}OH$, b) **UCY-16**/n-$C_8H_{17}OH$, c) **UCY-16**/n-$C_9H_{19}OH$, d) **UCY-16**/n-$C_{10}H_{21}OH$, e) **UCY-16**/n-$C_{12}H_{25}OH$, f) **UCY-16**/n-$C_{14}H_{29}OH$, g) **UCY-16**/n-$C_{16}H_{33}OH$ and h) digital photograph of a water droplet on a film of **UCY-16**/n-$C_{16}H_{33}OH$, along with the corresponding water contact angle measured using a contact angle goniometer.

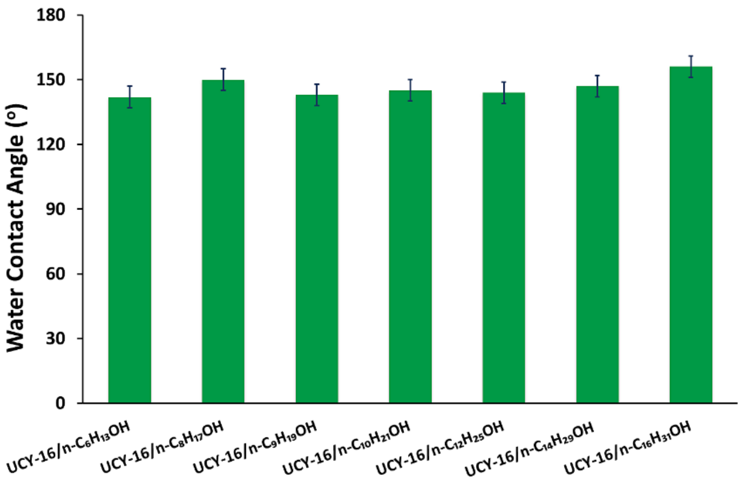

Figure S31: Water contact angle data for the exchanged analogues **UCY-16**/n-$C_xH_{2x+1}OH \cdot S'$ (x = 6, 8-10, 12, 14, 16) in a film form.

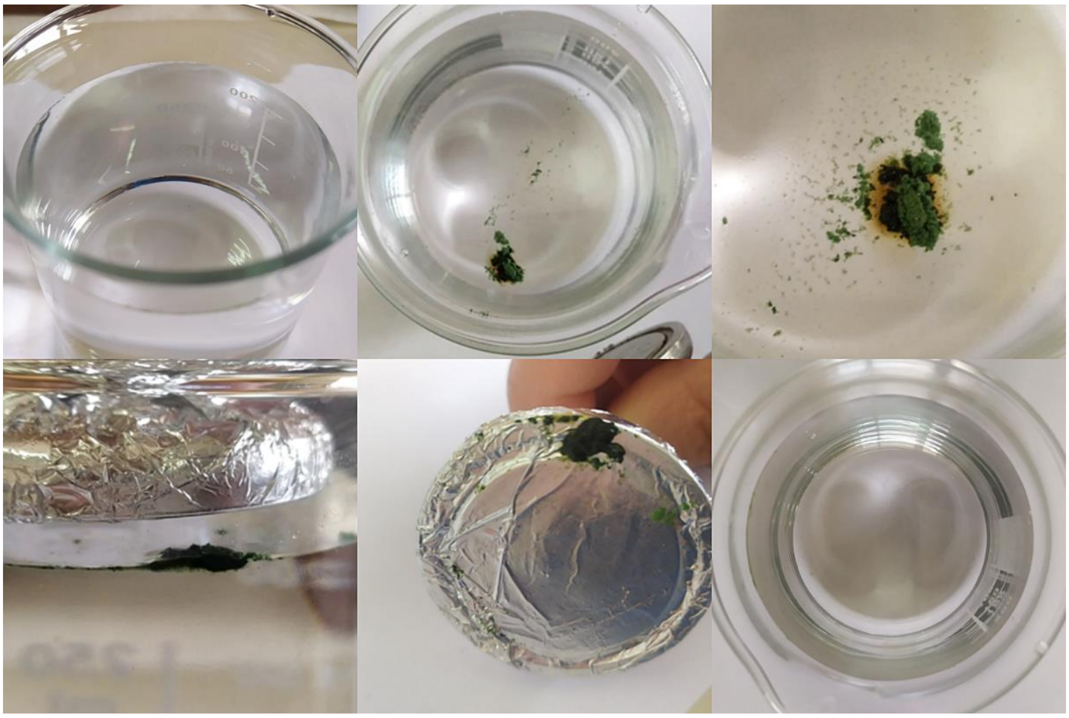

Figure S32: Crude oil removal by **UCY-16**/n-$C_{16}H_{33}OH/Fe_3O_4$ (mass ratio of 3:1) and recovery of the oil-laden material using an external magnet.
